# Psychometric evaluation of the 28-item coping orientation to problems experienced inventory (Brief COPE) amongst parents of preschool children from low-income backgrounds

**DOI:** 10.1016/j.ijnsa.2026.100625

**Published:** 2026-07-10

**Authors:** Jiying Ling, Dongjuan Xu, Yingcen Xie, Wen Liu

**Affiliations:** a MSU Research Foundation Distinguished Professor, Michigan State University College of Nursing, 1355 Bogue St., Room C241, East Lansing, MI, 48824, USA; b Associate Professor, Purdue University School of Nursing, USA; c Data Manager, Michigan State University College of Nursing, USA; d Associate Professor, Pennsylvania State University Ross and Carol Nese College of Nursing, USA

**Keywords:** Coping skills, Psychometrics, Factor analysis, Item response theory

## Abstract

**Background:**

The 28-item Coping Orientation to Problems Experienced Inventory (Brief COPE) is widely used to assess coping strategies, but reported factor structures range from 2 to 15 factors, raising concerns about structural stability. Only one study has applied Rasch analysis to examine unidimensionality and overall model fit of a four-factor French Brief COPE among cancer patients and caregivers. However, item fit and response categories were not evaluated.

**Objective:**

This study examined the psychometric properties of the Brief COPE among parents of preschoolers from low-income backgrounds. Specifically, we evaluated reliability using internal consistency and composite reliability; construct validity through structural validity, convergent validity, discriminant validity, and hypothesis testing with stress, anxiety, depression, and hair cortisol; and item- and response-option performance using item response theory analysis.

**Methods:**

We analyzed baseline data from three clinical trials conducted between 2021 and 2024. Participants were parents of preschoolers enrolled in Head Start programs in the Midwestern United States. Participants completed online surveys assessing coping, stress, anxiety, depression, and demographics, and provided hair samples for cortisol analysis.

**Results:**

The sample included 348 parents (*M_age_*=30.89 years), randomly divided for exploratory and confirmatory factor analysis. Exploratory factor analysis suggested a four-factor structure after removing four items due to high inter-item correlations or cross-loadings: adaptive coping, support coping, avoidant coping, and religious coping. Confirmatory factor analysis demonstrated good fit: CFI=0.92, TLI=0.91, RMSEA=0.06 (*90% CI*: 0.05–0.07), and SRMR=0.08. Convergent validity was adequate for support coping (average variance extracted=0.64) and religious coping (0.71), borderline for adaptive coping (0.49), and low for avoidant coping (0.38). Discriminant validity was supported by inter-construct correlations (*r*=0.10–0.59) and square roots of the average variance extracted values exceeding corresponding inter-construct correlations. Construct validity was partially supported by significant associations between coping dimensions and stress, anxiety, and depression, but not hair cortisol. Internal consistency (*α*=0.81–0.88) and composite reliability (0.83–0.90) were strong. Most items showed adequate difficulty and discrimination, while several appeared redundant. Information curves were unimodal. Response option “3″ was under-endorsed across multiple items, and several items had sparsely used options, indicating a need to collapse or revise response options.

**Conclusions:**

Findings support the reliability and validity of a four-factor, 24-item Brief COPE in parents of preschoolers with low-income. However, variability in item- and response-option performance indicates opportunities for targeted item consolidation and response-option refinement. Future efforts should implement these revisions and cross-validate the refined scale.

**What is already known**

 

• The Brief COPE is widely used to assess coping strategies across diverse populations and cultural contexts.
• Its factorial structure varies among populations and settings.
• To date, only one study has applied Rasch analysis to evaluate the unidimensionality and overall fit of a four-factor structure of the French Brief COPE among cancer patients and caregivers.

Alt-text: Unlabelled box dummy alt text

**What this paper adds**

 

• This study applied both classical test theory and item response theory to evaluate the psychometric properties of Brief COPE among parents of preschoolers with low income.
• Results support a four-factor structure comprising 24 items after removing four items with high inter-item correlations or cross-loadings.
• Most items demonstrated adequate difficulty and discrimination; however, some redundancy and underused response options indicate the need to revise or collapse response categories.

Alt-text: Unlabelled box dummy alt text

## 1 Background

Coping, a dynamic process of navigating efforts to manage perceived internal and external stressors, is essential for individuals to effectively respond to psychological stress and life challenges (Taylor and Stanton, 2007). A recent meta-analysis of 89 studies found that coping flexibility, the ability to adaptively apply different coping strategies based on situational demands, was linked to better psychological adjustments following major stressors (Chen et al., 2025). In contrast, maladaptive coping strategies have been directly associated with mental health problems (Groth et al., 2019). The central role of coping processes in mental health outcomes underscores the necessity of using reliable and valid measures of coping in both research and intervention contexts.

Parents from low-socioeconomic status disproportionally experience elevated levels of stress and mental health challenges (Marçal, 2024; Nagy et al., 2022). These parents often face unique, chronic life stressors including food insecurity, economic constraints, relationship difficulties, and disrupted parenting practices (Justice et al., 2025). Parenting during early childhood may be especially demanding because preschool-aged children are undergoing rapid social, emotional, behavioral, and cognitive development (Chopra, 2025). During this developmental period, parents may experience heightened stress related to children’s emotion regulation, behavior management, school readiness, social interactions, and daily caregiving routines (Williford et al., 2007). These challenges may differ from those faced by parents of adolescents or older children, whose parenting demands may involve different developmental tasks and stressors. Prior work suggests that parenting stress often decreases as children grow older (Mackler et al., 2015), further highlighting the importance of examining coping specifically among parents of preschoolers.

These poverty- and child development-related stressors compel parents to adopt a wide range of context-specific coping strategies to manage daily demands and care for their families (Mayo et al., 2022). For example, in response to food insecurity, families have been reported to apply various coping strategies such as seeking food assistance, altering purchasing behaviors, reducing food quality, and restricting food consumption (Byker Shanks et al., 2022). Similarly, among parents with infants, greater support seeking from families and friends has been related to lower stress (McKelvey et al., 2002).

While problem-focused coping strategies are frequently regarded as effective, some emotion-focused coping strategies such as emotional support or venting are found to buffer the adverse effects of perceived stress on chronic stress outcomes (Ling, Chen, and Marina, 2024). Moreover, in situations where stressors such as poverty and child development delays are beyond an individual’s control, emotion-focused coping can serve as an important temporary mechanism to alleviate emotional burden and preserve psychological resources. Reduced emotional burden and enhanced psychological resources, in turn, can enhance the capacity to engage in problem-focused coping when feasible (Carroll, 2020). These findings highlight the importance of understanding and conceptualizing the varied coping strategies among parents of preschoolers with low-socioeconomic status to inform future intervention efforts.

### 1.1 Brief COPE development and psychometric properties

The 28-item Coping Orientation to Problems Experienced Inventory (Brief COPE) is a widely used instrument for assessing adaptive and maladaptive coping strategies in adults (Carver, 1997). It was derived from the original 60-item COPE inventory that contains 14 subscales capturing diverse coping strategies (Carver et al., 1989). The measure is grounded in Lazarus and Folkman’s (1984) transactional model of stress and coping, which conceptualizes coping as cognitive and behavioral efforts to manage internal or external demands that are appraised as stressful, as well as Carver and Scheier’s (2012) self-regulation framework, which emphasizes goal-directed responses to stress. The COPE scale has two main formats: 1) a dispositional (trait-like) version with present-focused tense that asks what people usually do when stressed, and 2) a situational (state/event-specific) version with past- or present-focused tense that asks how people cope with a specific stressor during a particular period.

During refinement, Carver (1997) removed two poorly performing subscales (restraint and suppression of competing activities) and added a new subscale of *humor*. Thus, the Brief COPE includes 14 subscales: *active coping, use of informational support, positive reframing, planning, use of emotional support, venting, humor, acceptance, religion, self-blame, self-distraction, denial, substance use,* and *behavioral disengagement*. The Brief COPE was initially validated among 168 adults affected by a hurricane, and exploratory factor analysis yielded a nine-factor structure: *substance use, religion, humor, behavioral disengagement, social support, active coping/planning/positive reframing, venting/self-distraction, denial/self-blame, and acceptance* (Carver, 1997).

Subsequent research has revealed substantial variability in its factor structure, ranging from 2 to 15 factors across populations, raising concerns about its structural stability (Solberg et al., 2022). Among 85 studies included in a recent review, 47% tested the situational version, 28% did not specify, 22% focused on the dispositional version, and 2% examined both versions (Solberg et al., 2022). Across studies, the situational version yielded 2–14 factors (*mode*=2), while the dispositional version generated 2–8 factors (*mode*=3). Most studies retained all subscales, although about 22% removed one or more subscales due to poor psychometric performance: self-blame, substance use, self-distraction, and instrumental support (Solberg et al., 2022).

Parent-focused Brief COPE studies have also reported heterogeneous factor structures across populations, Brief COPE versions, and analytic approaches, with factor solutions ranging from 3 to 14 factors and varying item removal decisions (Benson, 2010; Hastings et al., 2005; Nunes et al., 2021; Steindorsdottir et al., 2024; Tang et al., 2021). These prior studies are summarized in Supplemental Table 1.

At the item level, we only identified one study that examined item-level psychometric models of the French Brief COPE among cancer patients and caregivers (Baumstarck et al., 2017). The study reported a four-factor structure (i.e., social support, problem solving, avoidance, positive thinking) and Rasch analysis confirmed the unidimensionality and good fit of each factor. However, the authors did not further examine the fit of individual items or the response categories.

### 1.2 Purpose

Although Brief COPE has demonstrated good internal consistency, evidence for its factorial structure, particularly the dispositional version, remains largely inconsistent across populations. The application of item-level models, such as item response theory, to examine its measurement precision or differential item functioning is very limited. Thus, the present study evaluated the psychometric properties of the 28-item Brief COPE dispositional version among parents of preschoolers from low-income backgrounds using classical test theory and item response theory. In particular, this study evaluated the reliability (composite reliability, internal consistency reliability) and construct validity (structural validity, convergent validity, discriminant validity) of the scale using classical test theory-based analyses as well as the item- and response option-level performance (item difficulty, item discrimination, threshold parameters, model fit, item/test information) using item response theory-based analyses in capturing coping behaviors within this high-risk population.

## 2 Methods

### 2.1 Data

The study used baseline data collected in three clinical trials conducted between 2021 and 2024 (Ling et al., 2025; Ling, Chen, et al., 2024, 2024). Parents completed an online survey through Qualtrics assessing their demographic characteristics, coping strategies, perceived stress, anxiety, and depression. In two of the trials (Ling, Chen, et al., 2024, 2024), a hair sample was collected from parents during in-person data collection visits for cortisol assay.

### 2.2 Sampling and eligibility criteria

In the prior clinical trials, participants were recruited nonrandomly through study flyers distributed in Head Start programs where teachers and administrators expressed interest in participating in the trials. Eligible participants were parents or legal guardians (hereafter, “parents”) of preschoolers aged 3–5 years enrolled in Head Start programs in Michigan, regardless of marital or partnership status. Parents with incomplete coping surveys were excluded from the present study’s analysis. In all clinical trials, parents were expected to participate in a health promotion intervention delivered through either a private Facebook group or study’s private website. Preschoolers were required to have no dietary restrictions that would prevent participation in eating behavior changes. In two clinical trials, preschoolers were also required to be able to participate in physical activities (Ling et al., 2025; Ling, Kao, et al., 2024). In one trial, preschoolers were excluded if they had severe communication difficulties (Ling et al., 2025).

### 2.3 Ethical considerations

The three clinical trials were approved by the local university institutional review board under approval IDs STUDY00001629, STUDY00003349, and STUDY00009256, respectively. In the clinical trials, parents provided informed consent electronically through Qualtrics before participating in research. The current study was also approved by the university institutional review board (STUDY202500783) to evaluate the psychometric properties of the Brief COPE using de-identified data. The dataset used in the study cannot be deposited in a public repository at this time because it is part of an ongoing study. Public release will occur once data collection has concluded, and all planned analyses have been completed. The COnsensus-based Standards for the selection of health Measurement INstruments reporting guidelines were followed in this manuscript (Gagnier et al., 2021).

### 2.4 Measures

#### 2.4.1 Demographic characteristics

Demographic questions were adapted from the National Health Interview Survey (National Center for Health Statistics, 2024) to assess dyads’ age, sex, and ethnicity/race, as well as parents’ marital status, employment status, education level, family annual income, and number of children living in each household.

#### 2.4.2 Coping strategies

The 28-item Brief COPE dispositional version was used to measure parents’ coping strategies (Carver, 1997; see Supplemental Table 2). The dispositional version was used because the study focused on parents’ usual coping strategies in daily life, rather than their coping responses to a specific stressful event. It is a 4-point Likert scale with response choices of *I haven’t been doing this at all, a little bit, a medium amount*, and *I’ve been doing this a lot*. It measured 14 different coping strategies with two items for each strategy (Carver, 1997). A higher sum score of the two items indicates greater use of the respective coping strategy.

#### 2.4.3 Perceived stress

The 10-item Perceived Stress Scale was utilized to assess parents’ perceived stress level (Cohen and Janicki-Deverts, 2012). Each item has five response choices including *never, almost never, sometimes, fairly often*, and *very often*. It has demonstrated good internal consistency reliability (> 0.70) and test-retest reliability (> 0.70), as well as criterion validity, evidenced by strong association with the mental health component of quality of life (Lee, 2012). It has also shown good known-groups validity, with lower perceived stress among participants who were younger, White, married, and employed. Overall, the scale has performed psychometrically better than the original 14-item Perceived Stress Scale (Lee, 2012). Evidence supports a two-factor structure: perceived helplessness and lack of self-efficacy (Taylor, 2015). A mean score was calculated, with a higher score indicating a greater level of perceived stress. In this study, Cronbach’s alpha was 0.87.

**Anxiety and depression** were assessed with the 4-item Patient Health Questionnaire (PHQ-4; Kroenke et al., 2009). Items were rated on a 4-point scale with responses ranging from *not at all* to *nearly every day*. Prior work in 5140 United States adults indicates a two-factor structure consisting of anxiety and depression, with strong internal consistency (Cronbach’s *α* = 0.92 for the total scale, 0.86 for anxiety, and 0.90 for depression; Adzrago et al., 2024). Its convergent validity was supported by significant associations with loneliness (*r* = 0.59–0.63) and sociodemographic characteristics, including age, sex, ethnicity, race, education level, marital status, employment status, and family annual income (Adzrago et al., 2024). We summed items to create a total score (range 0–12), with higher scores indicating greater symptom severity. In the present study, Cronbach’s alpha was 0.90 for the total scale, and 0.90 and 0.88 for the anxiety and depression subscales, respectively.

#### 2.4.4 Hair cortisol

Hair cortisol is a biomarker used to assess chronic hypothalamic-pituitary-adrenocortical activity and stress exposure over the past several weeks and months (Meyer and Novak, 2012). Prior evidence has shown that individuals in high-stress group have significantly higher hair cortisol levels than those in low-stress group (Huchegowda et al., 2025). Trained data collectors cut a small lock of hair (30–50 strands, 20 mg) from 2–3 sites at each parent’s posterior vertex using hair shears (Ling et al., 2024). Each sample was placed on aluminum foil with the proximal (scalp) end marked and then sealed in a labeled foil pouch. Labels included the parent’s study ID, data collection date, and study name. Samples were shipped to the University of Massachusetts at Amherst Hormone Assay Core (2022) and the Yale University Child Study Center Lab (2023–2024). In the laboratory, hair was washed twice in isopropanol (3 min each), dried, and grounded to a fine powder using a bead mill (BioSpec Mini-Beadbeater-16). Powdered hair was extracted in methanol for 18–24 h, and the methanolic extract was then evaporated and reconstituted in assay buffer. Cortisol concentrations were quantified by enzyme immunoassay (Arbor Assays DetectX). Mean intra- and inter-assay coefficients of variation were 11.8% and 7.7%, respectively, demonstrating acceptable precision.

### 2.5 Data analysis

Descriptive statistics were used to summarize the sample characteristics with IBM SPSS Statistics Version 30. The structural validity of the scale was examined using both exploratory factor analysis and confirmatory factor analysis with Stata 19. Item- and response option-level functioning was further evaluated using item response theory. Model fit was assessed using multiple indices, including the chi-square goodness-of-fit test, Comparative Fit Index (CFI ≥ 0.90 for confirmatory factor analysis, ≥ 0.95 for item response theory), Tucker–Lewis Index (TLI ≥ 0.90 for confirmatory factor analysis, ≥ 0.95 for item response theory), Root Mean Square Error of Approximation (RMSEA ≤ 0.08), and Weighted Root Mean Square Residual (WRMR < 1.0; Byrne, 2012; Hu and Bentler, 1999; Kline, 2011; Yu, 2002). These fit indices are mere guidelines and should not be interpreted as golden rules (Marsh et al., 2004).

Prior to factor analyses, bivariate correlations among items were examined. Item pairs with correlation coefficients ≥ 0.90 were considered highly redundant, and one item from each pair was evaluated for possible removal. The suitability of the data for factor analysis was assessed using the Kaiser-Meyer-Olkin measure of sampling adequacy and Bartlett’s test of sphericity. Kaiser-Meyer-Olkin values ≥ 0.60 were deemed acceptable, and a significant Bartlett’s test indicated that the correlation matrix was appropriate for factor analysis (Worthington and Whittaker, 2006).

Sample size adequacy for factor analysis was assessed using both the absolute sample size and the participant-to-item ratio. The initial scale included 28 items and was completed by 348 participants, yielding a participant-to-item ratio of 12.4:1 (6.2:1 when divided into two subsamples). This exceeded commonly cited minimum recommendations of at least 5 participants per item (Heckler, 1994) and also met the more conservative guideline of 10 participants per item (Nunnaly, 1978).

For factor analysis, the total sample was randomly divided into two comparable subsamples, one for exploratory factor analysis and the other for confirmatory factor analysis. In the first subsample, exploratory factor analysis was conducted using principal axis factoring with oblique rotation. The number of factors to be retained was determined using both the eigenvalue-greater-than-one criterion and parallel analysis (Osborne et al., 2008). Items with factor loadings < 0.30, or those exhibiting cross-loadings on two or more factors with loading differences of < 0.15, were considered candidates for removal (Worthington and Whittaker, 2006). The conceptual relevance of each item to the emerging factor structure was also reviewed. Items were removed when they demonstrated problematic cross-loadings and were conceptually less clear within the emerging factor structure.

After exploratory factor analysis and item retention, confirmatory factor analysis was performed on the second subsample. Standardized factor loadings ≥ 0.40 were regarded as acceptable indicators of the latent constructs (Brown, 2015). Composite reliability and average variance extracted were calculated using standardized factor loadings and error variances obtained from the confirmatory factor analysis. Composite reliability assesses the internal consistency of items measuring each latent construct, whereas average variance extracted evaluates convergent validity by estimating the proportion of item variance explained by the underlying construct. Composite reliability values greater than 0.70 indicate acceptable reliability, and average variance extracted values greater than 0.50 support adequate convergent validity (Fornell and Larcker, 1981).

Discriminant validity was evaluated using the Kline criterion and Fornell-Larcker criterion (Cheung and Wang, 2017; Fornell and Larcker, 1981). Kline criterion for discriminant validity is that the correlations between latent constructs are below 0.85. For each latent construct, the square root of the average variance extracted was compared with the correlations between that construct and the other latent constructs. Discriminant validity was supported when the square root of the average variance extracted for each construct exceeded its correlations with the other constructs, indicating that each construct shared more variance with its own indicators than with other latent constructs.

Following confirmatory factor analysis, internal consistency reliability was assessed for each retained factor and for the overall scale using Cronbach’s alpha and McDonald’s omega. Pearson correlation was performed to examine the correlations between coping strategies and theoretically relevant external variables, including perceived stress, anxiety, depression, and hair cortisol. The relations with external variables provided additional construct validity evidence.

Graded response models, two-parameter item response theory models for ordered polytomous responses, were estimated using Mplus Version 7.1 (Muthén and Muthén, 2010). The two-parameter model requires three assumptions: 1) unidimensionality (items in a [sub]scale measure only one underlying construct), 2) local independence (item responses are uncorrelated after accounting for the latent construct), and 3) minimal guessing in selecting response options (Rasch, 1960). Graded response models allow discrimination parameters to vary across response options for each item and are therefore more appropriate for the data than one-parameter models, which constrain discrimination to be equal across response options.

Sample size adequacy in 2-parameter item response theory models depends on the number of items and the number of response options per item. Although there is no standardized sample size requirement for item response theory analysis, research suggests a minimum of 250 to 500 respondents, at least 10 participants per item, and a minimum of 5 participants per response option for accurate parameter estimation (de Ayala, 2009; DeVellis, 2016). In the present study, item response theory models were estimated using a moderate sample size of 348 respondents across three confirmed factors consisting of nine, nine, and four items, respectively, with four response options per item. This yielded participant-to-item ratios of 38.7:1, 38.7:1, and 87.0:1, respectively, and participant-to-response-option ratios of 9.7:1, 9.7:1, and 21.8:1, respectively. These ratios met or exceeded the suggested minimum criteria.

Item performance was evaluated using item response theory parameters (item difficulties [locations] and discrimination) and graphical outputs (item characteristic curves, item information curves, and test information curves). Item difficulty represents the level of the latent construct associated with a 50% probability of endorsing a given response option and was estimated using the X-axis value corresponding to a 50% probability on the item characteristic curves (de Ayala, 2009; DeVellis, 2016). For items with three or more ordered response options, difficulty parameters are expected to increase across adjacent thresholds (de Ayala, 2009). Item discrimination estimates how well an item differentiates between individuals at different levels of the latent construct and was estimated using the slope of item characteristic curves: steeper slopes indicate better discrimination, whereas flat or negative slopes indicate poor discrimination (de Ayala, 2009; DeVellis, 2016).

Item characteristic curves demonstrate the relationship between the latent construct and the probability of endorsing each response choice. Item information curves show how much information each item provides across the latent construct continuum, independent of the other items in the (sub)scale. The test information curve aggregates information across all items within a (sub)scale, indicating the range over which the (sub)scale most effectively discriminates among individuals. Analysis codes are included in the supplemental materials.

## 3 Results

### 3.1 Participant characteristics

Data were collected from 348 parents, with a mean age of 30.89 years old (*SD* = 7.03). Among these parents, 6.0% identified as male, 6.9% as Hispanic, and 17.0% as Black. About 47.4% were single, 69.0% reported an annual family income below $30,000, and 44.3% were unemployed. In addition, more than half (52.4%) received a high school education or less. On average, each household included 2.65 children (*SD* = 1.40). Table 1 demonstrates the demographic characteristics of the participants.

**Table 1:** Demographic characteristics of parents (*N* = 348).

Continuous variables	*Mean*	*SD*
Age (years, range 19 - 69)	30.89	7.03
Number of children in family (range 1 - 9)	2.65	1.40
**Categorical variables**	***n***	***%***
Sex		
Male	21	6.0
Female	326	93.7
Other (non-binary)	1	0.3
Ethnicity (Hispanic)	24	6.9
Race		
White	245	70.4
Black	59	17.0
Asian	6	1.7
American Indian or Alaskan Natives	4	1.1
Multiracial	26	7.5
Other	5	1.4
Unknown/not reported	3	0.9
Marital status		
Married/partnered	156	44.8
Separated/divorced/widowed	27	7.8
Single	165	47.4
Family annual income		
Under $20,000	174	50.0
$20,000 - $29,999	66	19.0
$30,000 - $49,999	69	19.8
$50,000 or above	39	11.2
Employment status		
Full Time	108	31.0
Part Time	86	24.7
Unemployed	154	44.3
Education level		
Less than high school graduate	45	12.9
High school graduate	137	39.5
Some college	106	30.5
Technical school or community college degree	38	10.9
Bachelor's degree	17	4.9
Graduate or professional degree	5	1.4

***Notes***. *SD* = standard deviation; *n* = frequency; *%* = percentage.

### 3.2 Exploratory factor analysis

Bivariate correlations revealed a high correlation (*r* = 0.91) between COPE4 (“help me get through it”) and COPE11 (“make myself feel better”) regarding the use of alcohol or other drugs to cope. COPE11 was removed because its wording was more general and positive compared to COPE4. The Kaiser-Meyer-Olkin measure was 0.86, and Bartlett’s test of sphericity was significant (*p* < .001), indicating that the correlation matrix was appropriate for factor analysis. Both the eigenvalue-greater-than-one criterion and parallel analysis supported a four-factor solution.

The initial factor analysis indicated that three items showed problematic cross-loadings: COPE19 (“*I’ve been doing something to think about it less, such as going to movies, watching TV, reading, daydreaming, sleeping, or shopping*”) with loadings of 0.33 and 0.32; COPE21 (“*I’ve been expressing my negative feelings*”) with loadings of 0.32 and 0.30; and COPE28 (“*I’ve been making fun of the situation*”) with loadings of 0.43 and 0.35. These items were removed because they did not demonstrate clear factor membership and reduced the interpretability of the factor solution. Exploratory factor analysis was rerun with the remaining 24 items. The updated analysis yielded four factors: adaptive coping, support coping, avoidant coping, and religious coping (Table 2). The eigenvalues for the four factors were 5.51, 3.08, 1.35, and 1.09, respectively. All retained items loaded ≥ 0.30 on their respective factors without cross-loading issues (two items cross-loaded, but with loading differences > 0.23). Prior to rotation, the four-factor solution accounted for 92.4% of the common variance. Adaptive coping accounted for the largest proportion of common variance (46.2%), followed by support coping (25.8%), avoidant coping (11.3%), and religious coping (9.1%).

**Table 2:** Item-factor loadings from exploratory factor analysis (*n* = 174).

Item	Adaptive Coping	Avoidant Coping	Support Coping	Religious Coping	Uniqueness
	*λ*	*λ*	*λ*	*λ*	*u²*
**COPE2**. I've been concentrating my efforts on doing something about the situation I'm in.	.60				.60
**COPE7**. I've been taking action to try and make the situation better.	.71				.43
**COPE12**. I've been trying to see it in a different light, to make it seem more positive.	.46				.60
**COPE14**. I've been trying to come up with a strategy about what to do.	.79				.37
**COPE17**. I've been looking for something good in what is happening.	.55				.48
**COPE18**. I've been making jokes about it.	.37				.76
**COPE20**. I've been accepting the reality of the fact that it happened.	.66				.48
**COPE24**. I've been learning to live with it.	.48				.72
**COPE25**. I've been thinking hard about what steps to take.	.68				.46
**COPE1**. I've been turning to work or other activities to take my mind off things.		.30			.83
**COPE3**. I've been saying to myself "this isn't real."		.70			.50
**COPE4**. I've been using alcohol or other drugs to make myself feel better.		.36			.85
**COPE6**. I've been giving up trying to deal with it.		.67			.55
**COPE8**. I've been refusing to believe that it has happened.		.65			.47
**COPE9**. I've been saying things to let my unpleasant feeling escape.		.33			.82
**COPE13**. I've been criticizing myself.		.65			.44
**COPE16**. I've been giving up the attempt to cope.		.60			.64
**COPE26**. I've been blaming myself for things that happened.		.67			.47
**COPE5**. I've been getting emotional support from others.			.80		.43
**COPE10**. I've been getting help and advice from other people.			.76		.31
**COPE15**. I've been getting comfort and understanding from someone.			.73		.41
**COPE23**. I've been trying to get advice or help from other people about what to do.			.64		.43
**COPE22**. I've been trying to find comfort in my religion or spiritual beliefs.				.70	.45
**COPE27**. I've been praying or meditating.				.71	.45

***Notes***. *λ =* factor loading; uniqueness *u² =* the proportion of item variance not explained by the extracted factors.

### 3.3 Confirmatory factor analysis

**Fig. 1:**
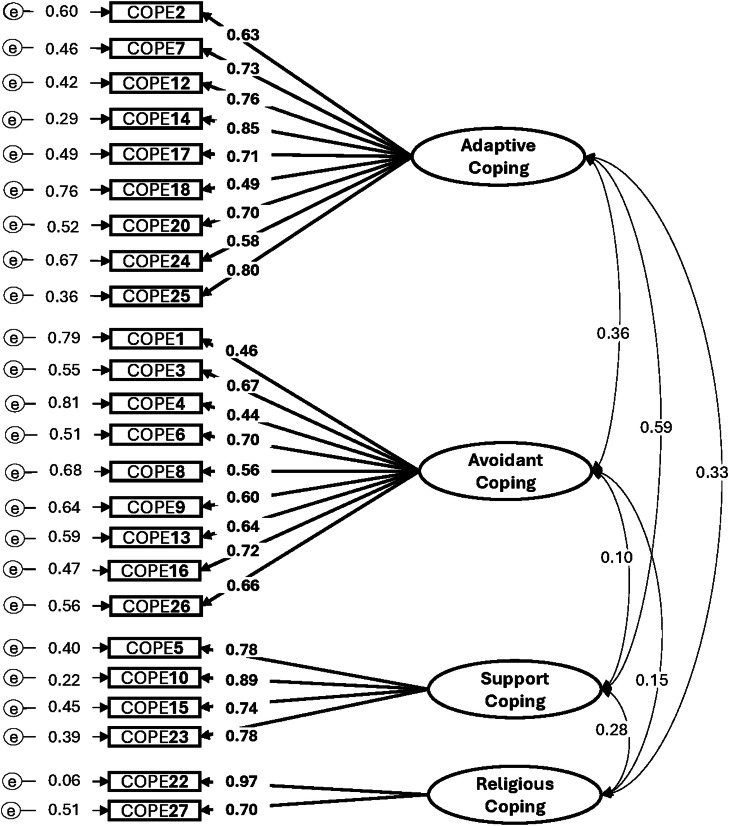
Factor loadings from confirmatory factor analysis (*n* = 174).

Confirmatory factor analysis was subsequently conducted on the second subsample. Results indicated that the four-factor structure provided a good fit to the data: CFI = 0.92, TLI = 0.91, RMSEA = 0.06 (*90% CI*: 0.05–0.07), and SRMR = 0.08. Standardized factor loadings for the 24 items were all ≥ 0.44, and the loadings for each item are presented in Fig. 1.

#### 3.3.1 Composite reliability and convergent validity

All constructs demonstrated good composite reliability, with values exceeding 0.80 for adaptive coping (0.90), avoidant coping (0.84), support coping (0.88), and religious coping (0.83). Average variance extracted values for support coping (0.64) and religious coping (0.71) exceeded the recommended threshold of 0.50, supporting satisfactory convergent validity. The average variance extracted value (0.49) for adaptive coping was borderline acceptable. In contrast, avoidant coping had a lower average variance extracted value (0.38), suggesting that some items, particularly COPE1 and COPE4, may share limited variance with the underlying construct despite acceptable internal consistency reliability.

#### 3.3.2 Discriminant validity

Inter-construct correlations ranged from 0.10 to 0.59, all below the 0.85 threshold. The square roots of the average variance extracted values were 0.70 for adaptive coping, 0.61 for avoidant coping, 0.80 for support coping, and 0.84 for religious coping, each exceeding its corresponding inter-construct correlations. The highest correlation was observed between adaptive coping and support coping (*r* = 0.59), which remained below the square root of the average variance extracted for both constructs, supporting adequate discriminant validity.

### 3.4 Hypothesis testing for construct validity

As demonstrated in Supplemental Table 3, adaptive coping was positively associated with perceived stress (*r* = 0.14, *p* = .009) and anxiety (*r* = 0.22, *p* = .005). Avoidant coping was strongly related to perceived stress (*r* = 0.75, *p* < .001), anxiety (*r* = 0.70, *p* < .001), and depression (*r* = 0.71, *p* < .001). Support coping showed a small correlation with perceived stress (*r* = 0.11, *p* = .049). Hair cortisol was not significantly correlated with any coping strategy; it showed a small, non-significant positive association with adaptive coping (*r* = 0.19, *p* = .070).

### 3.5 Internal consistency reliability

The Cronbach’s alpha for the remaining 24 items was 0.87 and McDonald’s omega was 0.86. Correlated item-total correlations ranged from 0.15 to 0.68. Cronbach’s alpha did not increase substantially when any single item was deleted, with a range of 0.86 to 0.87. The Cronbach’s alpha was 0.88, 0.81, 0.86, and 0.81 for the adaptive, avoidant, support, and religious coping, respectively.

### 3.6 Item response theory results

#### 3.6.1 Model fit

The Graded Response Models for the adaptive coping, avoidant coping, and support coping subscales produced mixed fit (Table 3). Across all models, high CFI and TLI values suggested acceptable model fit, whereas RMSEA and WRMR values indicated mediocre-to-good fit; however, the significant chi-square test results indicated poor model fit. An item response theory model was not estimated for the religious coping subscale because it included only two items.

**Table 3:** Criteria for item response theory-based model fit indices and model fit of each 1-factor structure (*N* = 348).

Model Fit Index	Model Fit Criteria	Adaptive Coping	Avoidant Coping	Support Coping
**χ² (*df*), p**	• Non-significance indicates good fit • Sensitive to large sample size, its significance should be interpreted with caution	144.960 (27) *p* < .001	108.786 (27) *p* < .001	25.911 (2) *p* < .001
RMSEA	• ≤ .08 indicate reasonable errors of approximation • .08 - .10 indicates mediocre fit	0.11	0.09	0.19
CFI	• ≥ .95 suggest an acceptable fit	0.97	0.96	0.99
TLI	• ≥ .95 suggest an acceptable fit	0.95	0.95	0.97
WRMR	• < 1.0 represents good model fit for categorical data • an experimental fit index, awaiting further testing of its properties	1.08	1.07	0.64

***Notes***. Item response theory model was not estimated for Factor 4 religious coping, because it only has two items. RMSEA = Root Mean Square Error of Approximation; CFI = Comparative Fit Index; TLI = Tucker–Lewis Index; WRMR = Weighted Root Mean Square Residual.

#### 3.6.2 Item difficulties (locations)

Standardized item locations for the three subscales are displayed in Table 4 and Supplemental Figure 1. Item location spanned the continua of adaptive coping (−1.81 to 2.06), avoidant coping (−1.37 to 5.12), and support coping (−1.11 to 1.38). Several adaptive coping items (COPE20, COPE25, and COPE14) clustered closely, suggesting potential redundancy. Avoidant coping items were more widely distributed but showed two clusters with minimum separation (COPE13 and COPE26; COPE3, COPE16, and COPE8). In addition, Step 2 and Step 3 thresholds overlapped for multiple items (COPE13, COPE9, COPE6, COPE16, and COPE8), indicating that the shift from “a little bit” to “a medium amount,” and from “a medium amount” to “a lot,” required similar levels of the latent trait, suggesting redundancy of the “a medium amount” option. The four items in the support coping subscale showed acceptable item difficulty levels.

**Table 4:** Item response theory-based model parameters for Brief COPE subscales (*N* = 348).

Adaptive Coping items	Std.a	*Std.*β		
		Step 1	Step 2	Step 3
**COPE2**	0.67	−1.77	−0.13	0.97
**COPE7**	0.78	−1.81	−0.52	0.50
**COPE12**	0.74	−1.40	0.02	1.03
**COPE14**	0.85	−1.30	−0.22	0.63
**COPE17**	0.74	−1.50	−0.21	0.82
**COPE18**	0.50	−0.43	0.97	2.06
**COPE20**	0.77	−1.36	−0.26	0.68
**COPE24**	0.65	−1.51	−0.05	0.95
**COPE25**	0.81	−1.34	−0.24	0.64
**Avoidant Coping items**				
**COPE1**	0.44	−1.37	0.78	2.33
**COPE3**	0.73	0.75	1.61	2.21
**COPE4**	0.54	2.22	3.92	5.12
**COPE6**	0.79	0.54	1.86	2.54
**COPE8**	0.74	0.89	1.92	2.45
**COPE9**	0.57	0.15	2.04	2.87
**COPE13**	0.82	−0.39	0.71	1.27
**COPE16**	0.81	0.83	1.96	2.31
**COPE26**	0.83	−0.24	0.68	1.30
**Support Coping Items**				
**COPE5**	0.81	−0.84	0.51	1.25
**COPE10**	0.91	−0.73	0.45	1.14
**COPE15**	0.79	−1.11	0.27	1.05
**COPE23**	0.81	−0.86	0.68	1.38

***Notes****. Std.* *a* = standardized step discrimination; *Std.*β= standardized item location (difficulty). Steps 1, 2, 3 represent the level of coping skills needed on the latent trait or underlying construct continuum (theta, θ e.g., attitude or ability level) where a respondent has a 50% probability of transitioning from one response category to the next (i.e., from response option 1 to 2, 2 to 3, and 3 to 4).

#### 3.6.3 Item discrimination

All items in the adaptive coping (0.50 to 0.85), avoidant coping (0.44 to 0.83), and support coping (0.79 to 0.91) had positive discrimination parameters, indicating that each item could distinguish participants on the latent traits (Table 4).

#### 3.6.4 Item characteristic curves

Item characteristic curves varied in location and shape based on item difficulties and discrimination parameters. For most items across all subscales, response category curves overlapped extensively with low peaks (Supplemental Figure 2), indicating that the response options were not well endorsed and provided limited discrimination. In particular, the response option “a medium amount” seemed redundant, because it was rarely or inconsistently endorsed, suggesting that collapsing or removing this option may improve scale performance. Item characteristic curves for COPE18 (“*I’ve been making jokes about it*”) in the adaptive coping subscale and many avoidant coping items (COPE1, 3, 4, 8, 9) were generally flat, reflecting poor discrimination and indicating a need for clearer item wording and more distinct response options.

#### 3.6.5 Item information curves

Item information curves (Supplemental Figure 3) showed varying contributions across items. For adaptive coping subscale, information curves were unimodal and symmetrical at around 0. COPE18 (“*I've been making jokes about it*”) provided the least information, while COPE14 (“*I've been trying to come up with a strategy about what to do*”) provided the most. Other items contributed moderate but varying levels of information.

For avoidant coping subscale, COPE1 (“*I've been turning to work or other activities to take my mind off things*”) and COPE4 (“*I've been using alcohol or other drugs to make myself feel better*”) provided the least information, with flat curves; and COPE13 (“*I've been criticizing myself*”) and COPE26 (“*I've been blaming myself for things that happened*”) provided the most information, with symmetrical curves around 0. Other items offered moderate information with peaks near 1.

For support coping subscale, information curves were unimodal and symmetrical at around 0. COPE10 (“*I've been getting help and advice from other people*”) provided the highest information, while other three items showed similar and overlapping curves.

#### 3.6.6 Test information curves

Test information curves for adaptive coping and support coping were unimodal and approximately symmetrical around 0, with peak information between −2.5 and 2.5 (Supplemental Figure 4), indicating good precision across a wide range of the latent trait. The avoidant coping subscale showed a unimodal but slightly asymmetrical curve, with peak information near 0.5 and high information between −1.5 and 2.5. Information at the extreme ends of all continua was limited, likely due to few participants endorsing very low or very high levels of the three constructs.

## 4 Discussion

This study applied classical test theory and item response theory to evaluate the 28-item Brief COPE dispositional version among 348 parents of young children from low-socioeconomic status backgrounds. The results support a modified four-factor structure with 24 retained items. The 24-item, four-factor structure demonstrated great model fit, strong internal consistency and composite reliability, and adequate discriminant validity. Convergent validity was adequate for support coping and religious coping, borderline for adaptive coping, and low for avoidant coping. Construct validity through hypothesis testing received partial support with some correlations with perceived stress, anxiety, and depression, but not hair cortisol. Results from item response theory suggest a need for further refinement, including targeted item consolidation and response-option adjustments. Future work should revise and revalidate items to further strengthen the scale’s psychometric properties.

The emergence of a 24-item, four-factor structure is broadly consistent with prior work showing that coping is multidimensional and may not be fully captured by the original two-item subscales or by a simple problem-focused versus emotion-focused distinction grounded in the Transactional Model of Stress and Coping (Lazarus and Folkman, 1984; Solberg et al., 2022). Together with the good fit observed in the confirmatory factor analysis, the results support the acceptability and structural coherence of the four-factor solution in the study’s population. The pattern of factors underscores theoretically meaningful distinctions (i.e., active problem-focused coping, approach-oriented support seeking, disengagement/avoidance, and faith-based coping) that can inform future research targeting parents of preschoolers from low-income backgrounds.

The revised Brief COPE may help identify coping patterns that could tailor support for parents. For example, parents reporting higher avoidant coping may need intervention components that focus on stress awareness, problem solving, emotion regulation, and reducing disengagement-based coping. Parents reporting lower support coping may benefit from strategies that strengthen social support, connect with family and peers, and access to community resources. The religious coping domain may also help identify faith-based or spiritually meaningful coping resources for families. Importantly, the revised Brief COPE should not be used as a diagnostic tool; rather, it may serve as a brief assessment to inform research, guide intervention tailoring, and evaluate changes in coping over time among parents facing socioeconomic and early-childhood parenting stressors.

Prior studies among parents of children with autism or other developmental conditions have also identified multidimensional coping structures, although the number and composition of factors have varied across samples (see Supplemental Table 1). For example, Hastings et al. (2005) identified four coping dimensions among parents of preschool- and school-age children with autism. Similarly, Benson (2010) reported four coping dimensions among mothers of children with autism. The avoidant coping factor identified in the present study is conceptually similar to the active avoidance, distraction, and disengagement dimensions reported in these prior studies, suggesting some cross-sample stability in avoidance-related coping responses.

The distinct support coping and religious coping factors identified in the present study suggest important differences in how coping strategies may cluster among parents of young children from low-income backgrounds. The identification of separate support and religious coping dimensions aligns with the original COPE framework, in which social support and religion were conceptualized as distinct coping domains (Carver et al., 1989). However, this pattern differs from some prior parent-focused studies, in which religious coping loaded with denial or was embedded within broader coping dimensions (Benson, 2010; Hastings et al., 2005). These differences suggest that support seeking and religious coping may function as more distinct coping resources in the present sample.

Variation in factor structure across studies may reflect differences in cultural context, parent populations, child age, child health or developmental conditions, and the types of stressors parents experience. For example, Tang et al. (2021) identified a three-factor structure (active coping, distraction, and dysfunctional coping) among Chinese parents of children with chronic illnesses, whereas Steindorsdottir et al. (2024) identified a six-factor structure among parents of children with and without learning disabilities in the United Kingdom, including external support-seeking, emotion-focused disengagement, positive cognitive reframing, substance use, religion, and problem-focused disengagement. These findings suggest that Brief COPE factor structures may be sensitive to sociocultural and contextual differences in how coping strategies are understood and used.

In this study, participants were parents of young children enrolled in Head Start programs, many of whom may experience stressors related to parenting demands, financial strain, and limited resources (Justice et al., 2025). These everyday stressors may differ from the condition-specific stressors experienced by parents of children with autism, learning disabilities, or chronic illnesses. As a result, coping strategies such as seeking support, relying on religion, reframing stressors, or avoiding stress-related thoughts may cluster differently in this population. Overall, the findings underscore the importance of evaluating the factor structure of coping measures within specific population and context in which they are used, rather than assuming that previously identified structures will generalize across cultural, socioeconomic, and caregiving contexts.

We removed four items based on redundancy and empirical misfit. The original COPE included only one alcohol/drug-use item (Carver et al., 1989); adding a second item on the same content may be unnecessary, as evidenced by the very high correlation in our data. COPE19 (self-distraction) showed problematic cross-loadings, consistent with prior reviews identifying self-distraction as one of the most frequently discarded subscales (Solberg et al., 2022). Regarding humor, which was added in the 1997 Brief COPE revision, COPE28 exhibited cross-loading, likely due to near-duplicate wording with COPE18, suggesting redundancy rather than a distinct facet. Finally, COPE21 was removed because of cross-loading, likely stemming from insufficiently specific behavioral target that does not clearly differentiate adaptive emotional expression from dysregulated venting, thus compromising construct clarity. The removal of COPE21 is consistent with a prior study showing that excluding the item improved internal consistency (Fernández-Martín et al., 2022). The removal of COPE21 and COPE28 is also supported by another study evaluating item-level discriminant content validity (Siaputra et al., 2023).

Composite reliability (0.83–0.90) was strong across all four factors, indicating adequate internal consistency at the latent construct level. Overall, composite reliability of the study’s four-factor structure appears stronger than that reported in a previous 14-factor structure with composite reliability ranging from 0.70 to 0.89 (Rodrigues et al., 2022). However, in contrast to the 14-factor structure showing acceptable convergent validity (Rodrigues et al., 2022), convergent validity in the present study was borderline for adaptive coping subscale and low for avoidant coping subscale. These appear to be driven in part by COPE18, COPE1, and COPE4, which had relatively low factor loadings on the related construct. Findings from the item response theory analysis further supported concerns about these items, as they demonstrated weak discrimination and contributed minimally to the underlying construct. These results suggest that COPE1, COPE4, and COPE18 may not perform optimally in this sample and that removing or revising these items may improve the psychometric performance of avoidant and adaptive coping subscales. Future work should evaluate whether item removal or modification improves convergent validity and overall model fit in independent samples.

Discriminant validity of the revised four-factor Brief COPE model was supported by both the Kline criterion and the Fornell-Larcker criterion, indicating that the four coping dimensions were empirically distinguishable. This finding is important because very few studies have formally evaluated the discriminant validity of Brief COPE. Rodrigues et al. (2022) reported adequate discriminant validity for the original 14-factor Brief COPE structure; however, our findings extend this evidence by demonstrating that a more parsimonious four-factor structure preserves conceptual and empirical distinctions among coping dimensions.

Compared with prior reports of internal consistency ranging from 0.37 to 0.89 (Benson, 2010; Nunes et al., 2021; Steindorsdottir et al., 2024; Tang et al., 2021), our final 24-item, four-factor structure demonstrated consistently higher reliability (0.81–0.88). Construct validity through hypothesis testing received partial support: adaptive coping was correlated with higher perceived stress and anxiety; avoidant coping showed robust positive associations with stress, anxiety, and depression; and support coping exhibited a small positive correlation with perceived stress. Because the data were cross-sectional, these associations should not be interpreted as evidence of causal effects of coping on mental health symptoms. Rather, the concurrent positive associations between coping and mental health symptoms may indicate that parents engage in more coping behaviors when experiencing greater psychological strain, consistent with the Transactional Model of Stress and Coping (Lazarus and Folkman, 1984). The relatively small associations of adaptive coping and support coping with mental health outcomes may also reflect less frequent use of these coping strategies in this sample (Hawken et al., 2018), rather than indicating that these strategies are ineffective. Religious coping was not associated with stress, anxiety, or depression. One plausible explanation is that the scale’s religious items combine passive (e.g., seeking comfort) and active (e.g., praying/meditating) forms of coping; prior work suggests active rather than passive religious coping relates more strongly to depressive symptoms (Aflakseir and Mahdiyar, 2016). Future efforts should focus on examining the predictive validity of Brief COPE using longitudinal data.

Hair cortisol was not significantly associated with any coping domain in the study. This lack of significant association may be partly due to the smaller subsample with available hair cortisol data (*n* = 93), which limited statistical power. However, adaptive coping showed a small positive correlation with hair cortisol, warranting further investigation in larger samples. This finding aligns with evidence in similar populations indicating that, among parents with relatively higher cortisol (i.e., more normative hypothalamic-pituitary-adrenal axis function), hair cortisol can be positively correlated with active coping (Ling et al., 2020). One possible interpretation is that hair cortisol, as an indicator of longer-term hypothalamic-pituitary-adrenal axis activity (Meyer and Novak, 2012), may reflect physiological stress-response capacity rather than stress burden alone. In this context, parents who engage in more adaptive or active coping may show a more responsive physiological stress system, whereas lower cortisol in chronically stressed populations may sometimes reflect hypothalamic-pituitary-adrenal axis downregulation or blunted stress physiology.

Overall, the improved reliability and the observed pattern of associations provide support for the revised Brief COPE structure in this sample. The findings highlight potentially context-specific links between coping dimensions and physiological stress, particularly for adaptive coping. Future studies with larger biomarker subsamples and longitudinal study designs are needed to replicate these findings and clarify how coping strategies relate to both perceived and physiological stress among parents of preschoolers from low-income backgrounds.

Findings from the item response theory analyses indicate that, although all remaining 24 items demonstrated adequate ability to discriminate among participants along their respective latent trait continua, varying degrees of item redundancy were evident within each subscale. This suggests that certain items may benefit from revision (e.g., COPE 1, 3, 4, 8, 9, 18), consolidation (e.g., COPE 20, 25 and 14; COPE 13 and 26; COPE3, 16, and 8), or removal (e.g., COPE 18, 1, 4). Additionally, across nearly all Brief COPE items, the response option “a medium amount” contributed little to differentiating participants, highlighting the need to either remove this option or refine at the response-option level. Among the three subscales, avoidant coping items showed the weakest performance, reflected in their item difficulty and discrimination estimates, as well as in the item characteristic curves, item information curves, and the total information curve. These converging results underscore the need for a more comprehensive evaluation and possible revision of the avoidant coping subscale at both the item and response option levels.

### 4.1 Limitations

This study has several limitations. First, the sample, parents (primarily mothers) of young children from households with low-socioeconomic status, limits external validity. Findings may not generalize to families with higher-socioeconomic status or other parent/child age groups. Second, the religious coping subscale included only two items, limiting its suitability for factor-specific item response theory analysis. Because at least three items per factor are generally preferred for stable factor-level psychometric evaluation, item response theory analysis was not conducted for the religious coping subscale. Third, criterion-related validity could not be established because there is no gold standard for assessing coping strategies. Our evidence is therefore limited to internal structure and convergent associations. Lastly, the cross-sectional design precludes causal inference and prevents evaluation of predictive validity or temporal stability. Future work should test measurement invariance and generalizability in more diverse samples and utilize longitudinal designs (with test-retest and responsiveness analyses) to assess predictive performance. Where feasible, multi-method and multi-informant approaches can be employed to mitigate shared-method bias.

## 5 Conclusion

This study is the first to apply both classical test theory and item response theory to evaluate the Brief COPE among parents of preschoolers from low-income backgrounds, providing item-level evidence that complements factor-analytic findings. The resulting 24-item, four-factor structure demonstrated strong fit and reliability, offering a concise, psychometrically supported framework for assessing coping strategies in this population. Findings also pinpoint specific targets for refinement, such as selective item consolidation and response-option adjustments, to further enhance measurement validity. The revised measure may be applied to examine coping patterns, identifying parents who may benefit from targeted support, and evaluating changes in coping over time. Future research should conduct confirmatory testing in diverse populations, systematically revise and revalidate selected items, and evaluate the predictive validity of the revised Brief COPE using longitudinal study designs.

## Publication ethics

The study was approved by the Michigan State University Institutional Review Board (STUDY202500783). Informed consent was obtained from all participating parents.

### Funding

Data used in the study were collected with support from the National Center for Complementary and Integrative Health (UG3/UH3AT012521, PI: J Ling), National Institute of Nursing Research (R21NR017958, PI: J Ling), and Michigan Health Endowment Fund (G-1904–144,322, PI: J Ling). The contents are solely the responsibility of the authors and do not necessarily represent the official views of any funding agency.

### Open science

The authors are willing to share their data, analytics methods, and study materials with other researchers. The material will be available upon request from the corresponding author Dr. Jiying Ling at lingjiyi@msu.edu.

## CRediT authorship contribution statement

**Jiying Ling:** Writing – review & editing, Writing – original draft, Supervision, Project administration, Methodology, Investigation, Funding acquisition, Formal analysis, Data curation, Conceptualization. **Dongjuan Xu:** Writing – original draft, Methodology, Formal analysis. **Yingcen Xie:** Writing – original draft, Formal analysis, Data curation. **Wen Liu:** Writing – review & editing, Writing – original draft, Methodology, Formal analysis.

## Declaration of competing interest

The authors declare that they have no known competing financial interests or personal relationships that could have appeared to influence the work reported in this paper.

## Appendix Supplementary materials

Image, application 1
