# Supplementary material for: Psychometric evaluation of the 28-item coping orientation to problems experienced inventory (Brief COPE) amongst parents of preschool children from low-income backgrounds
Source: Int J Nurs Stud Adv. 2026 Jul 10;11:100625. doi: 10.1016/j.ijnsa.2026.100625 (PMC13393768; doi:10.1016/j.ijnsa.2026.100625)
Supplement: Supplementary file 1 [file mmc1.docx]

**Supplemental Table 1. Summary of prior psychometric studies of the Brief COPE relevant to the current study**

| Study | Participants | Sample size | Brief COPE version | Analytic approach | Factor structure or main results | Item removal or item-level results | Reliability |
| --- | --- | --- | --- | --- | --- | --- | --- |
| Carver, 1997 | Adults affected by a hurricane | 168 | Brief COPE; original validation | Exploratory factor analysis with oblique rotation | Nine-factor structure: substance use, religion, humor, behavioral disengagement, social support, active coping/planning/positive reframing, venting/self-distraction, denial/self-blame, and acceptance | Brief COPE refined from original COPE; restraint and suppression of competing activities removed; self-blame added | Cronbach’s α = 0.50–0.90 |
| Solberg et al., 2022 | Systematic review | 85 studies | Both | Systematic review | Factors ranged from 2 to 15; situational version yielded 2-14 factors; dispositional version yielded 2-8 factors | About 22% of studies removed items or subscales, most commonly self-blame, substance use, self-distraction, and instrumental support | Varied across studies |
| Benson, 2010 | Mothers of school age children with autism, United States | 113 | Situational version | Exploratory factor analysis with varimax rotation | Four factors: engagement, distraction, disengagement, and cognitive reframing | No removal | Cronbach’s α = 0.73–0.86 |
| Hastings et al., 2005 | Parents of preschool and school age children with autism, United Kingdom | 135 | Situational version | Exploratory factor analysis with varimax rotation | Four factors: active avoidance coping, problem-focused coping, positive coping, and religious/denial coping | Two items on self-distraction and acceptance were excluded | Cronbach’s α = 0.68–0.82 |
| Tang et al., 2021 | Asian parents of school age children with chronic illness | 217 | Chinese dispositional version | Exploratory factor analysis with oblique rotation | Three factors: active coping, dysfunctional coping, and distraction | No removal | Cronbach’s α = 0.57–0.89 |
| Steindorsdottir et al., 2024 | Parents of children aged 5–18 years with and without learning disabilities, United Kingdom | 175 | Situational version | Exploratory factor analysis | Six factors: external support-seeking, emotion-focused disengagement, positive cognitive reframing, substance use, religion, and problem-focused disengagement | Self-distraction item 1 was excluded | Cronbach’s α > 0.70 for all factors |
| Nunes et al., 2021 | Parents of school age children, Portugal | 269 | Portuguese version | Confirmatory factor analysis | Supported the original 14-factor structure | No removal | Cronbach’s α = 0.37–0.88 |
| Baumstarck et al., 2017 | Cancer patients and caregivers, France | 398 | French Brief COPE dispositional version | Exploratory factor analysis with varimax rotation, Rasch analysis | Four factors: social support, problem solving, avoidance, and positive thinking; Rasch analysis supported unidimensionality and overall fit of each factor | No removal, did not evaluate individual item fit or response categories | Cronbach’s α = 0.71–0.82 |

**Factor analyses codes in Stata**

use "U:\ Brief_COPE.dta" ,clear

pwcorr T1_P_cope4 T1_P_cope11 if RandomGroup ==0

tab T1_P_cope4 T1_P_cope11 if RandomGroup ==0

//**EFA**//

/* not including T1_P_cope11 */

factor T1_P_cope2 T1_P_cope7 T1_P_cope12 T1_P_cope14 T1_P_cope17 T1_P_cope18 T1_P_cope19 T1_P_cope20 T1_P_cope21 T1_P_cope24 T1_P_cope25 T1_P_cope1 T1_P_cope3 T1_P_cope4 T1_P_cope6 T1_P_cope8 T1_P_cope9 T1_P_cope13 T1_P_cope16 T1_P_cope26 T1_P_cope28 T1_P_cope5 T1_P_cope10 T1_P_cope15 T1_P_cope23 T1_P_cope22 T1_P_cope27 if RandomGroup ==0, pf

screeplot

factor T1_P_cope2 T1_P_cope7 T1_P_cope12 T1_P_cope14 T1_P_cope17 T1_P_cope18 T1_P_cope19 T1_P_cope20 T1_P_cope21 T1_P_cope24 T1_P_cope25 T1_P_cope1 T1_P_cope3 T1_P_cope4 T1_P_cope6 T1_P_cope8 T1_P_cope9 T1_P_cope13 T1_P_cope16 T1_P_cope26 T1_P_cope28 T1_P_cope5 T1_P_cope10 T1_P_cope15 T1_P_cope23 T1_P_cope22 T1_P_cope27 if RandomGroup ==0, pf factor(4)

rotate , promax

/* not including T1_P_cope11, T1_P_cope19, T1_P_cope21, and T1_P_cope28 */

factor T1_P_cope2 T1_P_cope7 T1_P_cope12 T1_P_cope14 T1_P_cope17 T1_P_cope18 T1_P_cope20 T1_P_cope24 T1_P_cope25 T1_P_cope1 T1_P_cope3 T1_P_cope4 T1_P_cope6 T1_P_cope8 T1_P_cope9 T1_P_cope13 T1_P_cope16 T1_P_cope26 T1_P_cope5 T1_P_cope10 T1_P_cope15 T1_P_cope23 T1_P_cope22 T1_P_cope27 if RandomGroup ==0, pf factor(4)

rotate , promax

//**CFA**//

sem (F1 -> T1_P_cope2 T1_P_cope7 T1_P_cope12 T1_P_cope14 T1_P_cope17 T1_P_cope18 T1_P_cope20 T1_P_cope24 T1_P_cope25 , ) (F2 -> T1_P_cope1 T1_P_cope3 T1_P_cope4 T1_P_cope6 T1_P_cope8 T1_P_cope9 T1_P_cope13 T1_P_cope16 T1_P_cope26 , ) (F3 -> T1_P_cope5 T1_P_cope10 T1_P_cope15 T1_P_cope23 , ) (F4 -> T1_P_cope22 T1_P_cope27, ) if RandomGroup ==1, cov(F1*F2 F1*F3 F1*F4 F2*F3 F2*F4 F3*F4) cov(e.T1_P_cope13*e.T1_P_cope26) cov(e.T1_P_cope12*e.T1_P_cope25) cov(e.T1_P_cope4*e.T1_P_cope23) cov(e.T1_P_cope14*e.T1_P_cope18) cov(e.T1_P_cope7*e.T1_P_cope22) cov(e.T1_P_cope6*e.T1_P_cope16) cov(e.T1_P_cope6*e.T1_P_cope8) cov(e.T1_P_cope8*e.T1_P_cope9) cov(e.T1_P_cope2*e.T1_P_cope22) cov(e.T1_P_cope2*e.T1_P_cope24) cov(e.T1_P_cope24*e.T1_P_cope3) cov(e.T1_P_cope24*e.T1_P_cope15) cov(e.T1_P_cope12*e.T1_P_cope23) method(ml) latent(F1 F2 F3 F4 ) nocapslatent

estat gof, stats(all)

sem, standardized

**Item Response Theory analysis codes in Mplus**

TITLE: COPE factor 1 - 9 items_Likert_2PL Graded Response MODEL_Mplus(n=348)

DATA: File is "C:\Desktop\factor1-9 items_4cat.dat";

VARIABLE: Names are COPE1 - COPE9;

Categorical Are COPE1 - COPE9;

ANALYSIS: ESTIMATOR = WLSMV;

Model: COPE BY COPE1 - COPE9 *;

COPE@1;

OUTPUT: STDYX Residual;

TECH1 TECH8;

SAVEDATA: SAVE = FSCORES;

FILE IS COPE_42Thetas.dat;

Plot: Type = plot1 plot2 plot3;

TITLE: COPE factor 2 - 9 items_Likert_2PL Graded Response MODEL_Mplus(n=348)

DATA: File is "C:\Desktop\factor2-9 items_4cat.dat";

VARIABLE: Names are COPE1-COPE9;

Categorical Are COPE1 - COPE9;

ANALYSIS: ESTIMATOR = WLSMV;

Model: COPE BY COPE1 - COPE9 *;

COPE@1;

OUTPUT: STDYX Residual;

TECH1 TECH8;

SAVEDATA: SAVE = FSCORES;

FILE IS COPE_42Thetas.dat;

Plot: Type = plot1 plot2 plot3;

TITLE: COPE factor 3 - 4 items_Likert_2PL Graded Response MODEL_Mplus(n=348)

DATA: File is "C:\Desktop\factor3-4 items_4cat.dat";

VARIABLE: Names are COPE1-COPE4;

Categorical Are COPE1 - COPE4;

ANALYSIS: ESTIMATOR = WLSMV;

Model: COPE BY COPE1 - COPE4 *;

COPE@1;

OUTPUT: STDYX Residual;

TECH1 TECH8;

SAVEDATA: SAVE = FSCORES;

FILE IS COPE_42Thetas.dat;

Plot: Type = plot1 plot2 plot3;

**Supplemental Table 2.** The 28-item Brief COPE (*N* = 348)

| **Item** | **Not at all**  ***n (%)*** | **A little bit**  ***n (%)*** | **A medium amount**  ***n (%)*** | **A lot**  ***n (%)*** |
| --- | --- | --- | --- | --- |
| **COPE1**. I've been turning to work or other activities to take my mind off things. | 96 (27.6) | 124 (35.6) | 74 (21.3) | 54 (15.5) |
| **COPE2**. I've been concentrating my efforts on doing something about the situation I'm in. | 41 (11.8) | 121 (34.8) | 96 (27.6) | 90 (25.9) |
| **COPE3**. I've been saying to myself "this isn't real." | 246 (70.7) | 60 (17.2) | 23 (6.6) | 19 (5.5) |
| **COPE4**. I've been using alcohol or other drugs to make myself feel better. | 308 (88.5) | 34 (9.8) | 5 (1.4) | 1 (0.3) |
| **COPE5**. I've been getting emotional support from others. | 86 (24.7) | 144 (41.4) | 64 (18.4) | 54 (15.5) |
| **COPE6**. I've been giving up trying to deal with it. | 231 (66.4) | 92 (26.4) | 17 (4.9) | 8 (2.3) |
| **COPE7**. I've been taking action to try and make the situation better. | 27 (7.8) | 92 (26.4) | 108 (31.0) | 121 (34.8) |
| **COPE8**. I've been refusing to believe that it has happened. | 259 (74.4) | 62 (17.8) | 15 (4.3) | 12 (3.4) |
| **COPE9**. I've been saying things to let my unpleasant feeling escape. | 186 (53.4) | 119 (34.2) | 25 (7.2) | 18 (5.2) |
| **COPE10**. I've been getting help and advice from other people. | 88 (25.3) | 141 (40.5) | 67 (19.3) | 52 (14.9) |
| **COPE11**. I've been using alcohol or other drugs to help me get through it. | 316 (90.8) | 27 (7.8) | 4 (1.1) | 1 (0.3) |
| **COPE12**. I've been trying to see it in a different light, to make it seem more positive. | 52 (14.9) | 124 (35.6) | 95 (27.3) | 77 (22.1) |
| **COPE13**. I've been criticizing myself. | 131 (37.6) | 119 (34.2) | 46 (13.2) | 52 (14.9) |
| **COPE14**. I've been trying to come up with a strategy about what to do. | 47 (13.5) | 101 (29.0) | 97 (27.9) | 103 (29.6) |
| **COPE15**. I've been getting comfort and understanding from someone. | 66 (19.0) | 137 (39.4) | 74 (21.3) | 71 (20.4) |
| **COPE16**. I've been giving up the attempt to cope. | 260 (74.7) | 68 (19.5) | 9 (2.6) | 11 (3.2) |
| **COPE17**. I've been looking for something good in what is happening. | 46 (13.2) | 107 (30.7) | 101 (29.0) | 94 (27.0) |
| **COPE18**. I've been making jokes about it. | 144 (41.4) | 95 (27.3) | 57 (16.4) | 52 (14.9) |
| **COPE19**. I've been doing something to think about it less, such as going to movies, watching TV, reading, daydreaming, sleeping, or shopping. | 83 (23.9) | 124 (35.6) | 66 (19.0) | 75 (21.6) |
| **COPE20**. I've been accepting the reality of the fact that it happened. | 52 (14.9) | 95 (27.3) | 96 (27.6) | 105 (30.2) |
| **COPE21**. I've been expressing my negative feelings. | 109 (31.3) | 161 (46.3) | 49 (14.1) | 29 (8.3) |
| **COPE22**. I've been trying to find comfort in my religion or spiritual beliefs. | 161 (46.3) | 91 (26.1) | 36 (10.3) | 60 (17.2) |
| **COPE23**. I've been trying to get advice or help from other people about what to do. | 85 (24.4) | 162 (46.6) | 55 (15.8) | 46 (13.2) |
| **COPE24**. I've been learning to live with it. | 57 (16.4) | 113 (32.5) | 85 (24.4) | 93 (26.7) |
| **COPE25**. I've been thinking hard about what steps to take. | 48 (13.8) | 99 (28.4) | 96 (27.6) | 105 (30.2) |
| **COPE26**. I've been blaming myself for things that happened. | 147 (42.2) | 101 (29.0) | 51 (14.7) | 49 (14.1) |
| **COPE27**. I've been praying or meditating. | 127 (36.5) | 90 (25.9) | 46 (13.2) | 85 (24.4) |
| **COPE28**. I've been making fun of the situation. | 216 (62.1) | 80 (23) | 28 (8) | 24 (6.9) |

**Supplemental Table 3**. Concurrent correlations among coping, stress, anxiety, depression, and hair cortisol

| **Variable** | Adaptive coping | Avoidant coping | Support coping | Religious coping | Perceived stress | Anxiety |
| --- | --- | --- | --- | --- | --- | --- |
| Avoidant coping (*n*=348) | .31** |  |  |  |  |  |
| Support coping (*n*=348) | .49** | .15* |  |  |  |  |
| Religious coping (*n*=348) | .30** | .09 | .22** |  |  |  |
| Perceived stress (*n*=348) | .14* | .75** | .11* | -.01 |  |  |
| Anxiety (*n*=168) | .22* | .70** | .10 | .02 | .72** |  |
| Depression (*n*=168) | .14 | .71** | -.05 | .02 | .73** | .68** |
| Hair cortisol (*n*=93) | .19 | .07 | -.02 | -.04 | -.06 | – |

Notes. **p*<.05, ***p*<.001

| **Supplemental Figure 1.** Standardized item location parameters for Brief COPE Factor 1 Adaptive Coping (Figure 1a), Factor 2 Avoidant Coping (Figure 1b), and Factor 3 Support Coping (Figure 1c) |
| --- |
| \| COPE7 \| COPE2 \| COPE24 \| COPE17 \| COPE12 \| COPE20 \| COPE25 \| COPE14 \| COPE18 \| \| --- \| --- \| --- \| --- \| --- \| --- \| --- \| --- \| --- \|   **Figure 1a.** Adaptive Coping |
| \| COPE1 \| COPE13 \| COPE26 \| COPE9 \| COPE6 \| COPE3 \| COPE16 \| COPE8 \| COPE4 \| \| --- \| --- \| --- \| --- \| --- \| --- \| --- \| --- \| --- \|   **Figure 1b.** Avoidant Coping |
|  |

| \| COPE15 \| COPE23 \| COPE5 \| COPE10 \| \| --- \| --- \| --- \| --- \|   **Figure 1c.** Support Coping | |
| --- | --- | --- | --- | --- | --- |
| ***Notes.*** The Y-axis shows the standardized item difficulties (locations). Items are scored such that frequency (high score) reflected higher or lower levels of applying coping strategies in participants. Steps 1, 2, 3 represent the level of coping skills needed on the latent trait or underlying construct continuum (theta, $\theta$ e.g., attitude or ability level) where a respondent has a 50% probability of transitioning from one response category to the next (i.e., from response option 1 to 2, 2 to 3, and 3 to 4). |  |

**Supplemental Figure 2.** Items Characteristic Curves for Brief COPE Factor 1 Adaptive Coping (Figure 2a), Factor 2 Avoidant Coping (Figure 2b), and Factor 3 Support Coping (Figure 2c)

| **COPE2**  **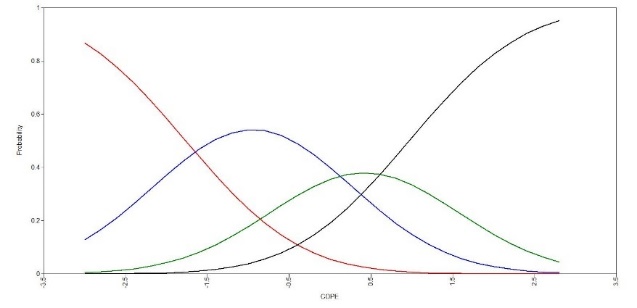** | **COPE7**  **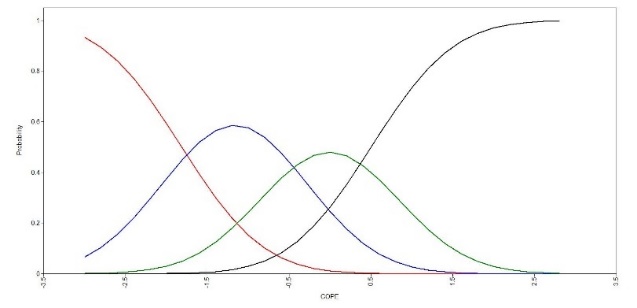** |
| --- | --- |
| **COPE12**  **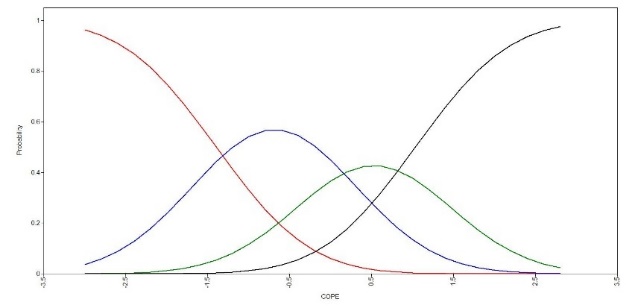** | **COPE14**  **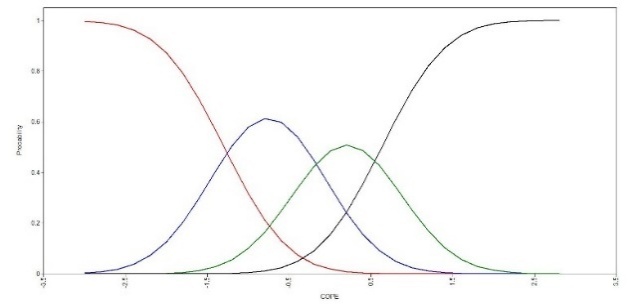** |
| **COPE17**  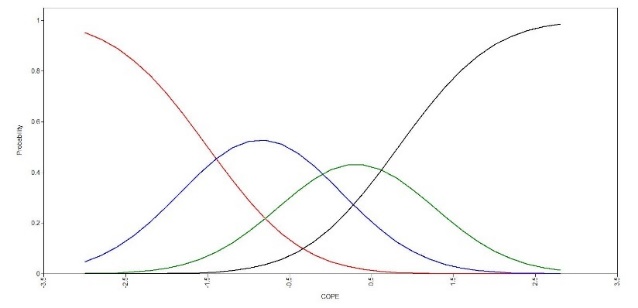 | **COPE18**  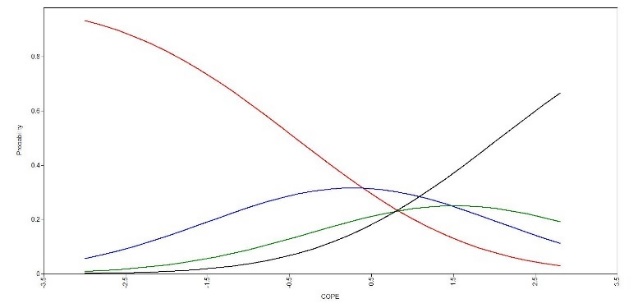 |
| **COPE20**  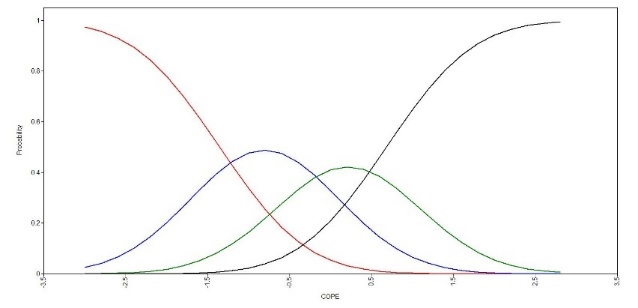 | **COPE24**  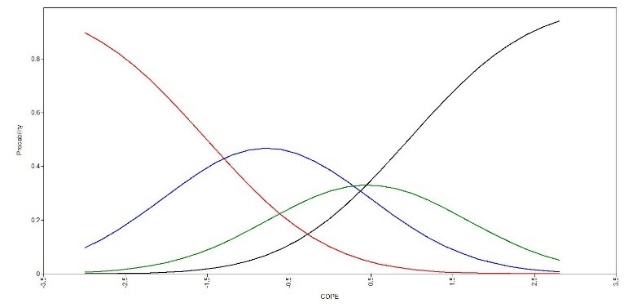 |
| **COPE25**  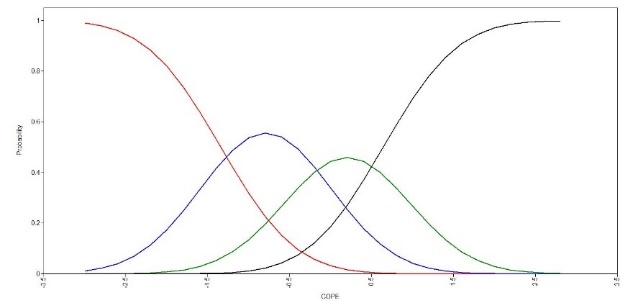 | \| *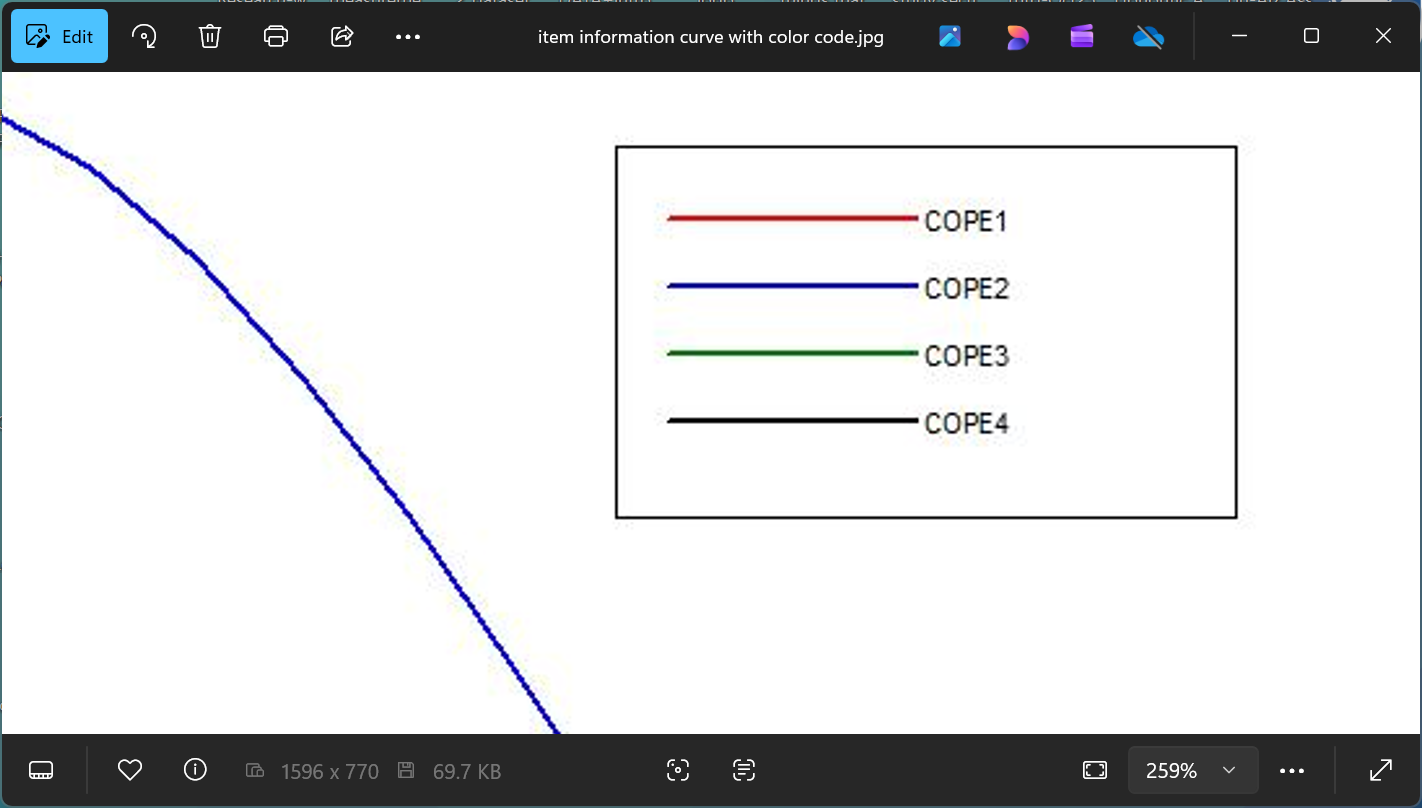* \| Response option #1: Not at all \| \| --- \| --- \| \| Response option #2: A little bit \| \| Response option #3: A medium amount \| \| Response option #4: A lot \| |
| **Figure 2a.** Adaptive Coping | |
| **COPE1**  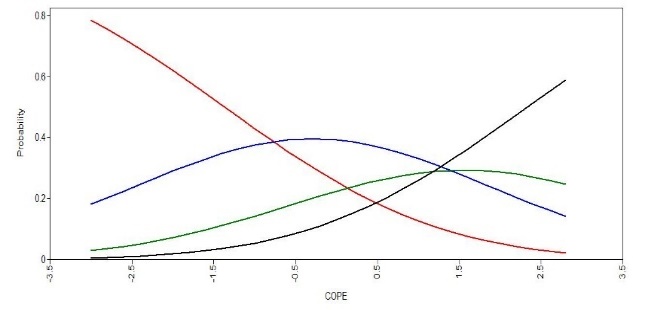 | **COPE3**  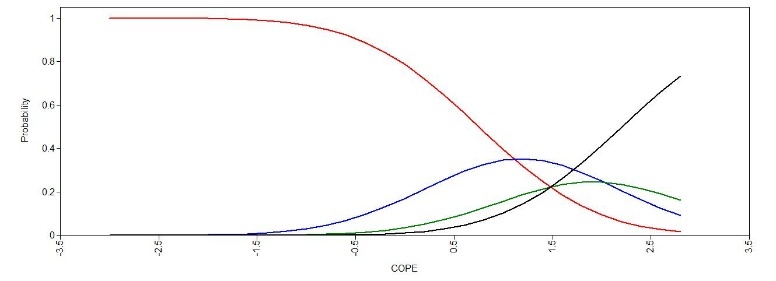 |
| **COPE4**  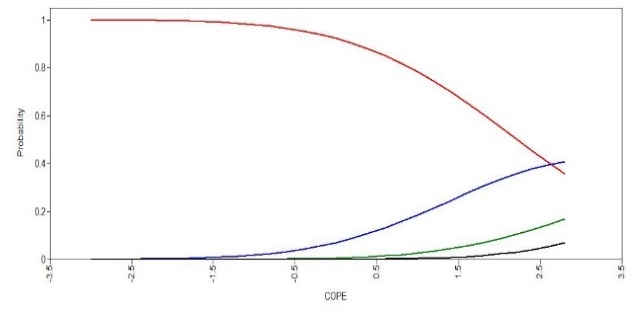 | **COPE6**  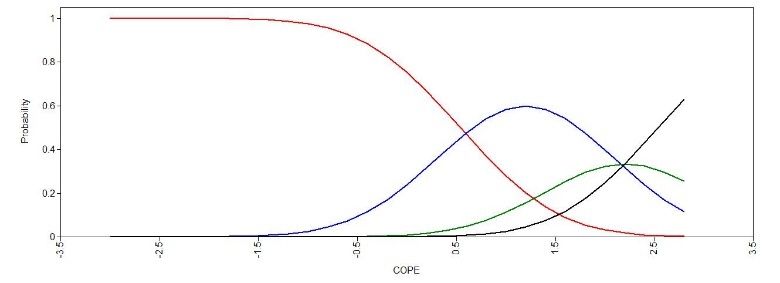 |
| **COPE8**  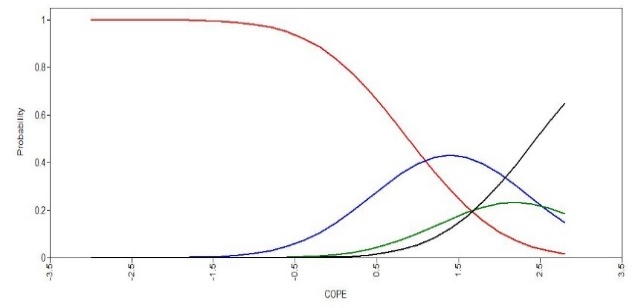 | **COPE9**  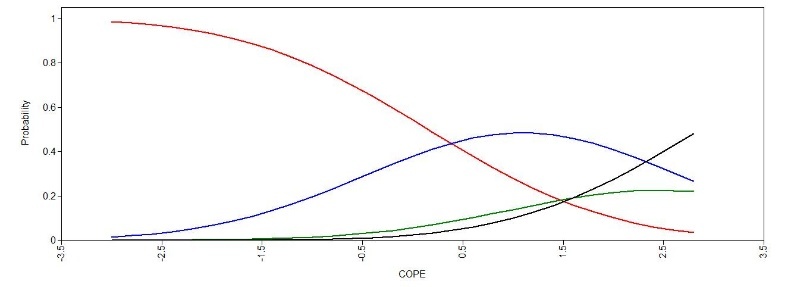 |
| **COPE13**  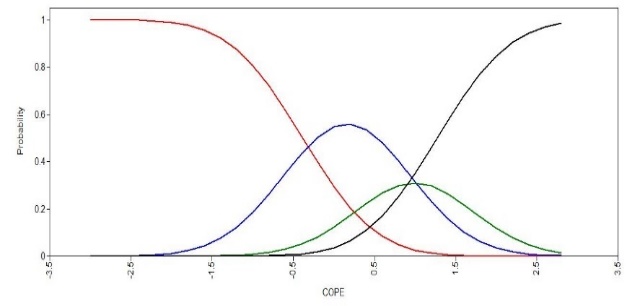 | **COPE16**  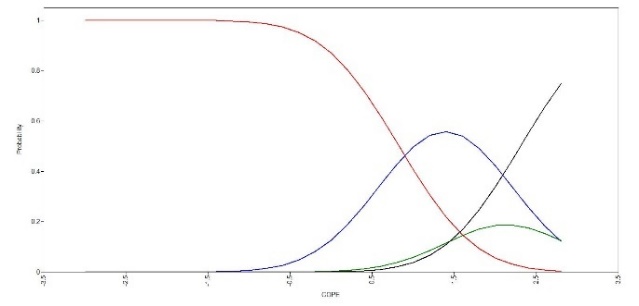 |
| **COPE26**  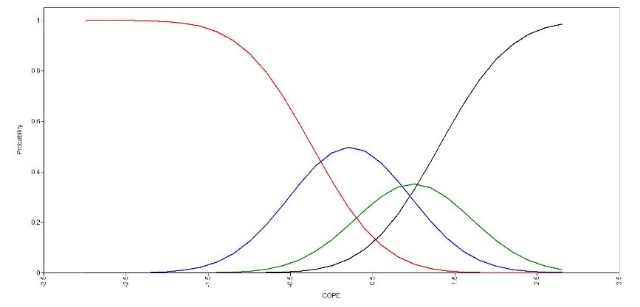 | \| *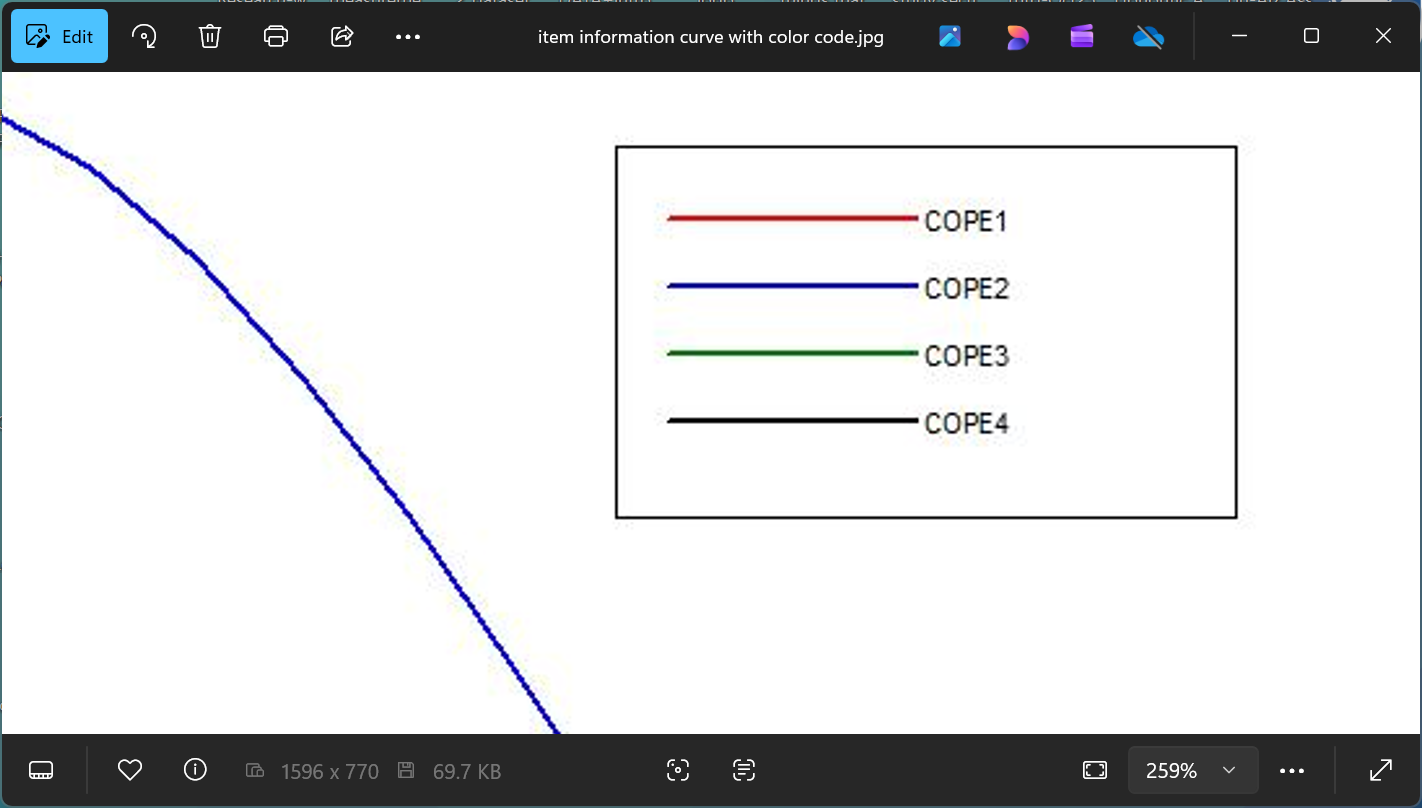* \| Response option #1: Not at all \| \| --- \| --- \| \| Response option #2: A little bit \| \| Response option #3: A medium amount \| \| Response option #4: A lot \| |
| **Figure 2b.** Avoidant Coping | |
|  | |
| **COPE5**  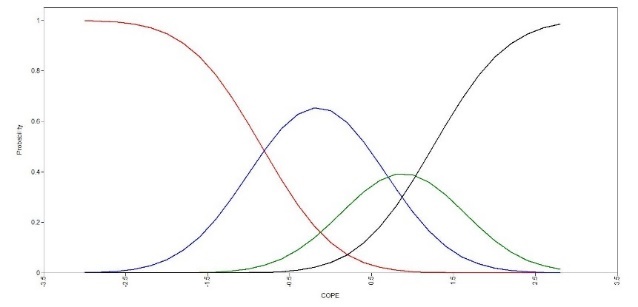 | **COPE10**  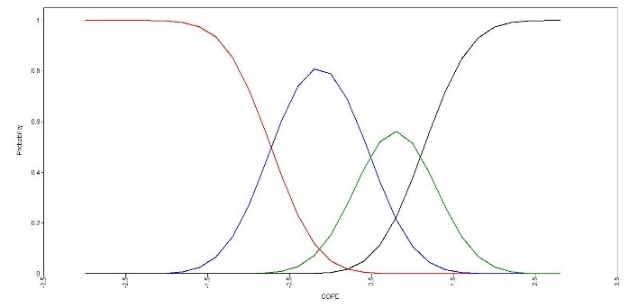 |
| **COPE15**  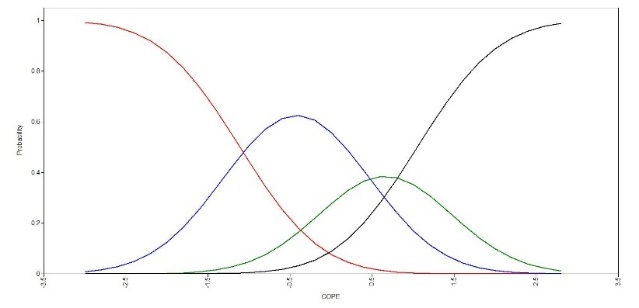 | **COPE23**  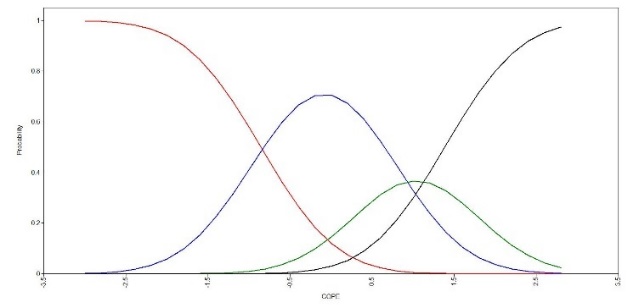 |
| \| *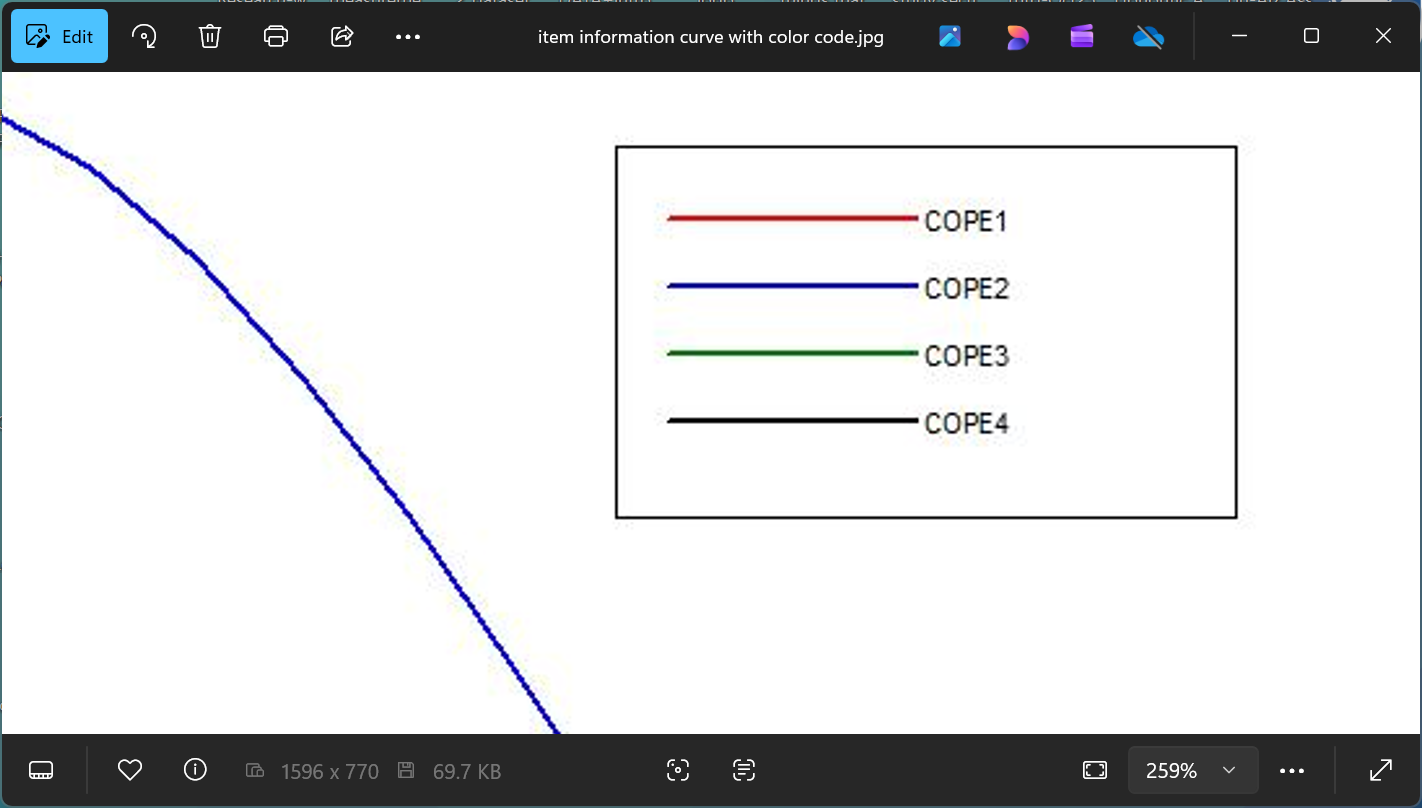* \| Response option #1: Not at all \| \| --- \| --- \| \| Response option #2: A little bit \| \| Response option #3: A medium amount \| \| Response option #4: A lot \|   **Figure 2c.** Support Coping | |

***Notes***. Figures showing the performance of items in each factor with four response options (1-4). The x-axis indicates the latent trait or underlying construct continuum (theta, $\theta)-a$daptive coping (Figure 2a), avoidant coping (Figure 2b), and support coping (Figure 2c). Solid lines show predicted probabilities for each response along the continuum of the latent trait.

| **Supplemental Figure 3.** Item Information Curves for Brief COPE Factor 1 Adaptive Coping (Figure 3a), Factor 2 Avoidant Coping (Figure 3b), and Factor 3 Support Coping (Figure 3c)   \| *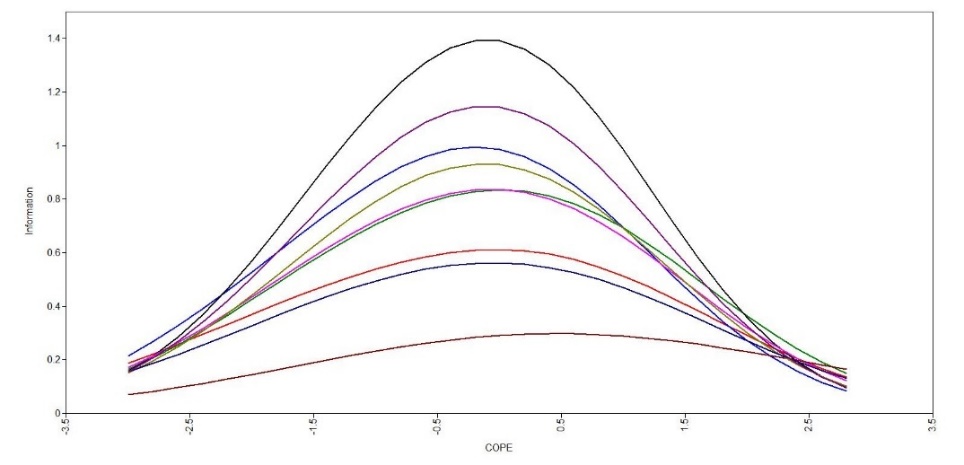* \| \| *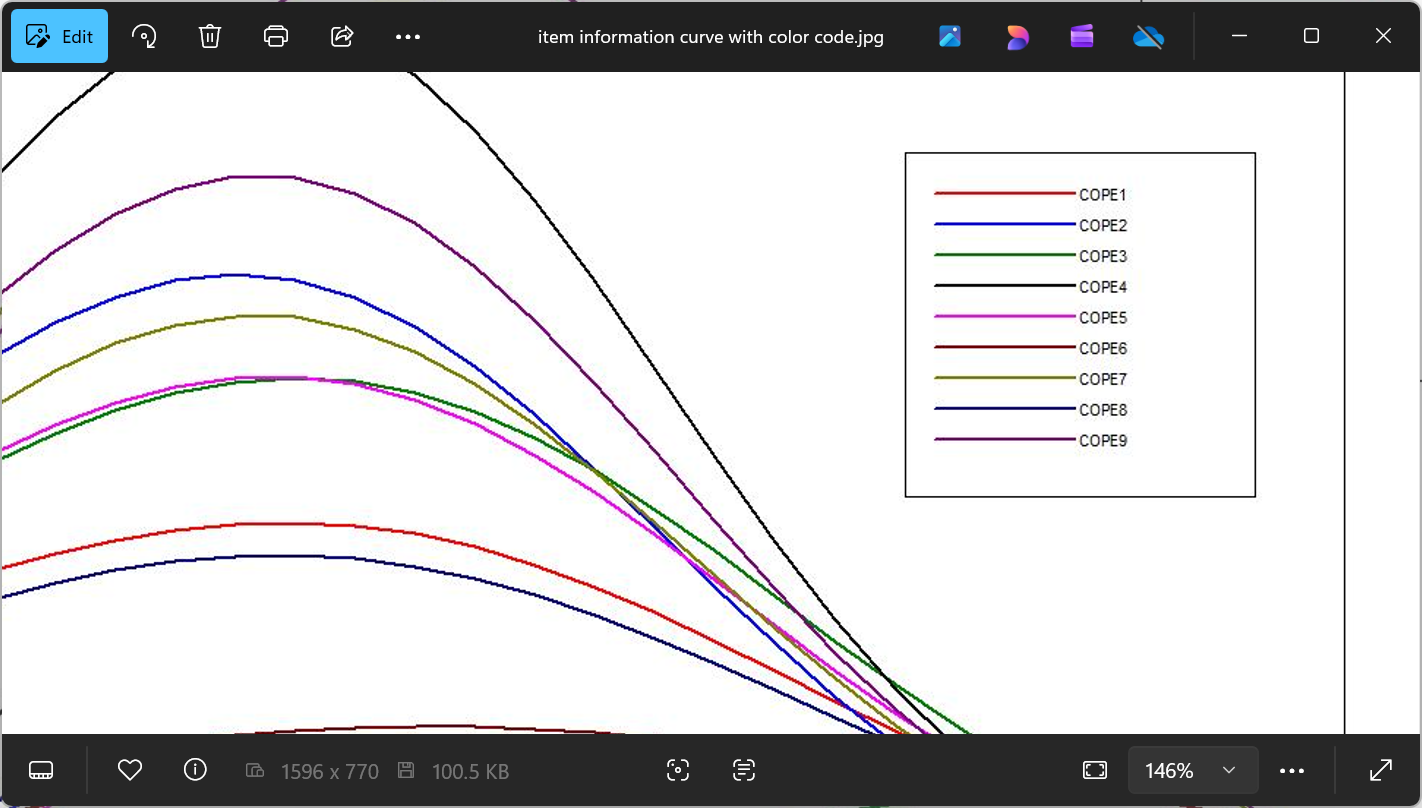* \| COPE2 \| \| --- \| --- \| \| COPE7 \| \| COPE12 \| \| COPE14 \| \| COPE17 \| \| COPE18 \| \| COPE20 \| \| COPE24 \| \| COPE25 \| \| \| --- \| --- \| --- \| --- \| --- \| --- \| --- \| --- \| --- \| --- \| --- \| --- \| |  |  |
| --- | --- | --- | --- | --- | --- | --- | --- | --- | --- | --- | --- | --- | --- | --- |
| **Figure 3a**. Adaptive Coping | |  |
|  | |  |
| \| \| *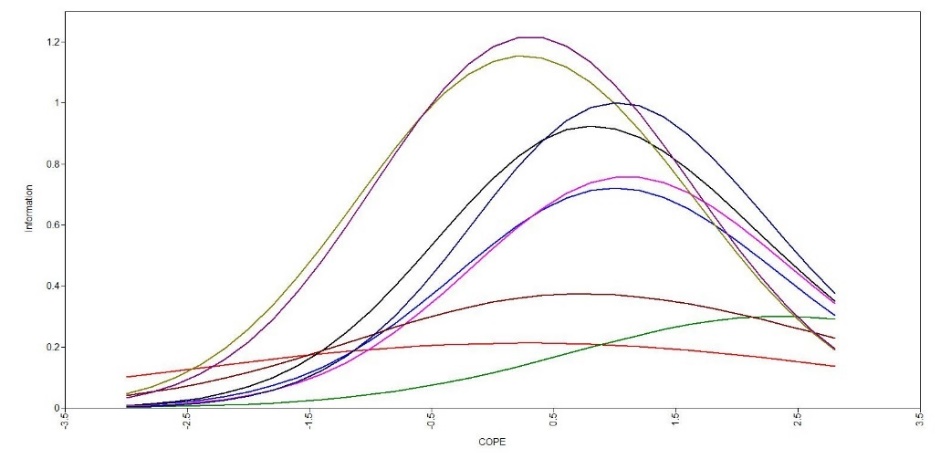* \| \| *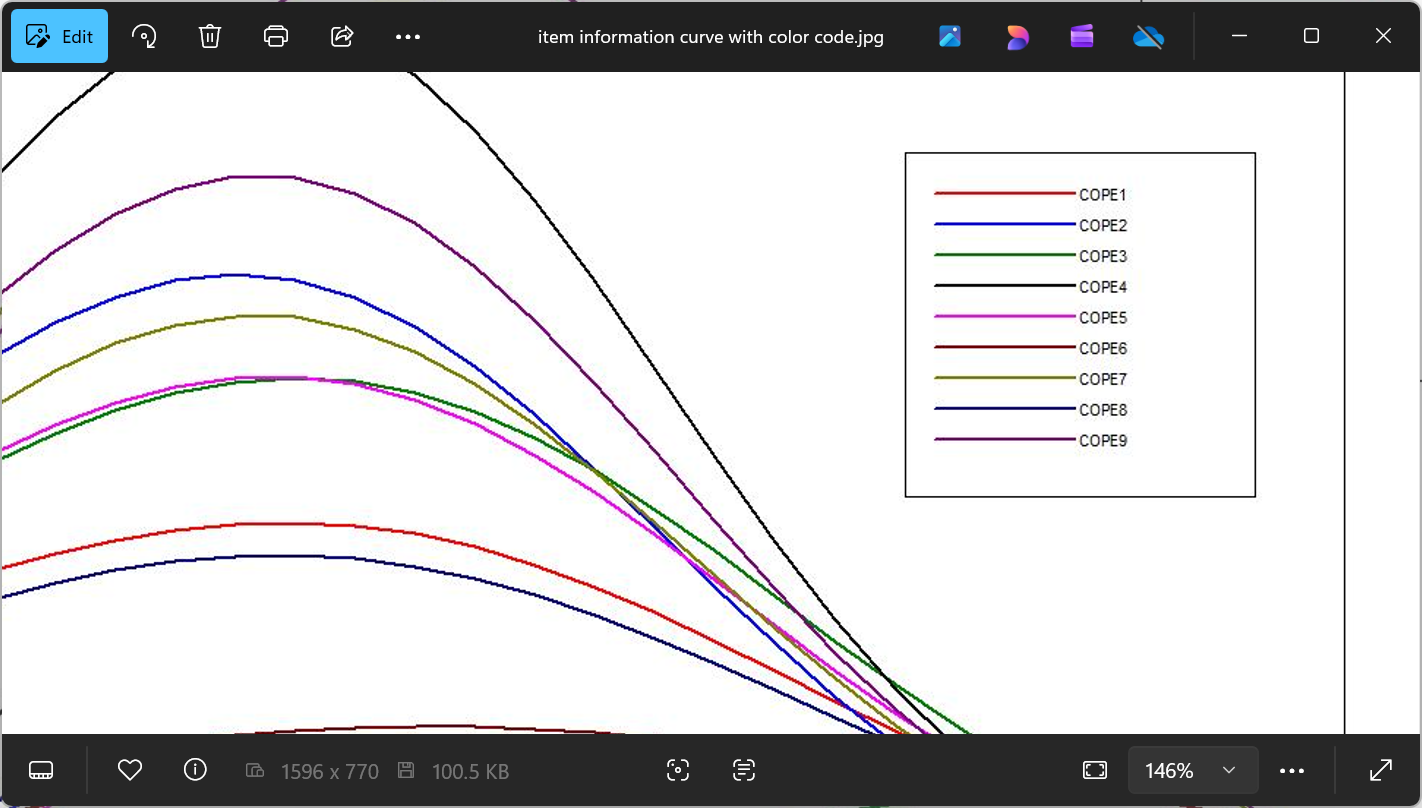* \| COPE1 \| \| --- \| --- \| \| COPE3 \| \| COPE4 \| \| COPE6 \| \| COPE8 \| \| COPE9 \| \| COPE13 \| \| COPE16 \| \| COPE26 \| \| \| --- \| --- \| --- \| --- \| --- \| --- \| --- \| --- \| --- \| --- \| --- \| --- \| \| \| \| \| --- \| --- \| --- \| --- \| --- \| --- \| --- \| --- \| --- \| --- \| --- \| --- \| --- \| --- \| --- \| \| **Figure 3b**. Avoidant Coping \|  \| \|  \|  \| \| \| \| *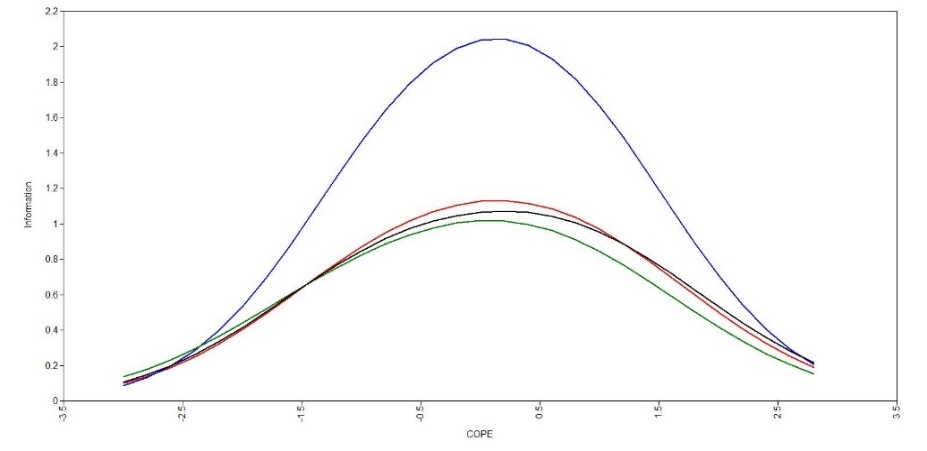* \| \| *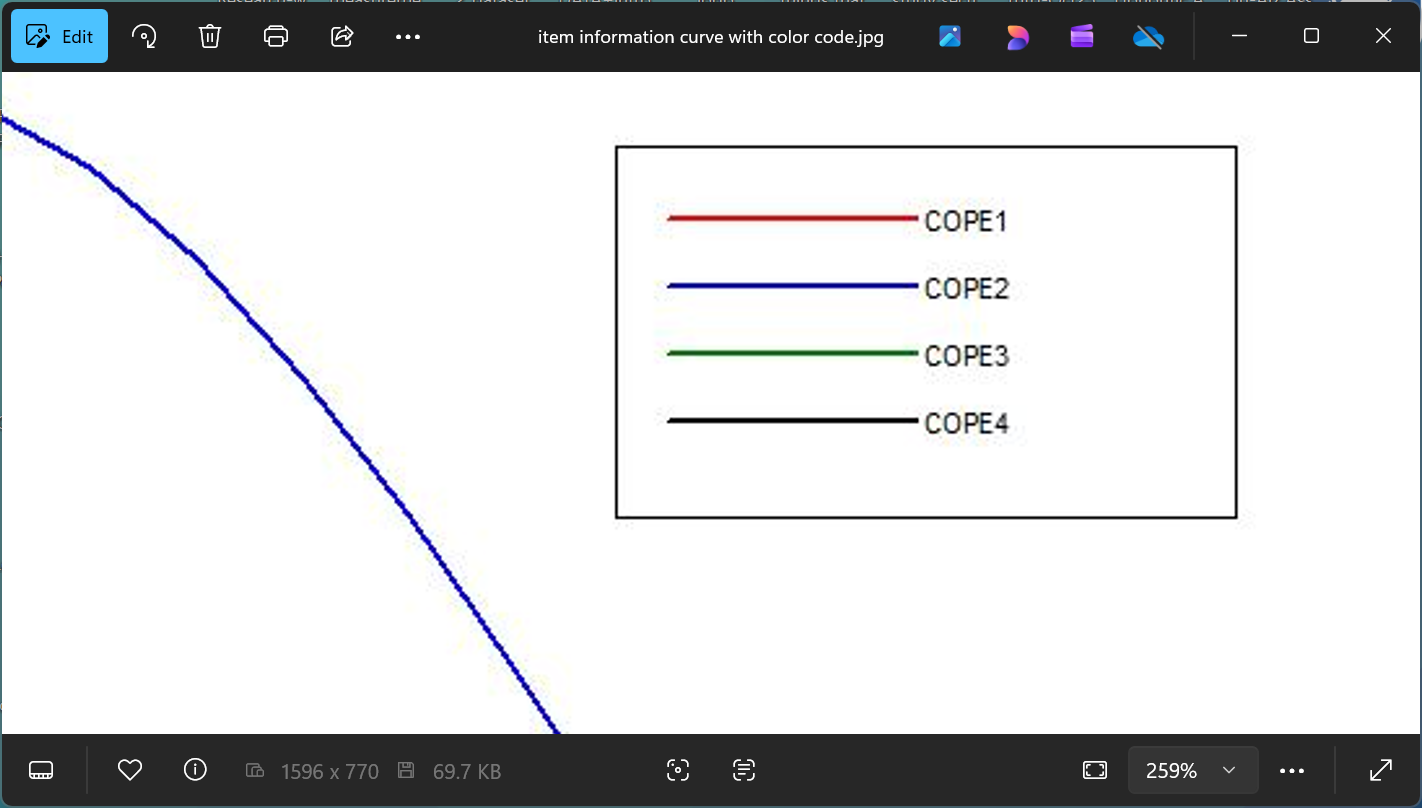* \| COPE5 \| \| --- \| --- \| \| COPE10 \| \| COPE15 \| \| COPE23 \| \| \| --- \| --- \| --- \| --- \| --- \| --- \| --- \| \| \| \| --- \| --- \| --- \| --- \| --- \| --- \| --- \| --- \| --- \| \| **Figure 3c**. Support Coping \|  \| \|  \|  \| \| \| \| | |  |

| **Supplemental Figure 4.** Test information function for Brief COPE Factor 1 Adaptive Coping (Figure 4a), Factor 2 Avoidant Coping (Figure 4b), and Factor 3 Support Coping (Figure 4c) | |
| --- | --- |
| 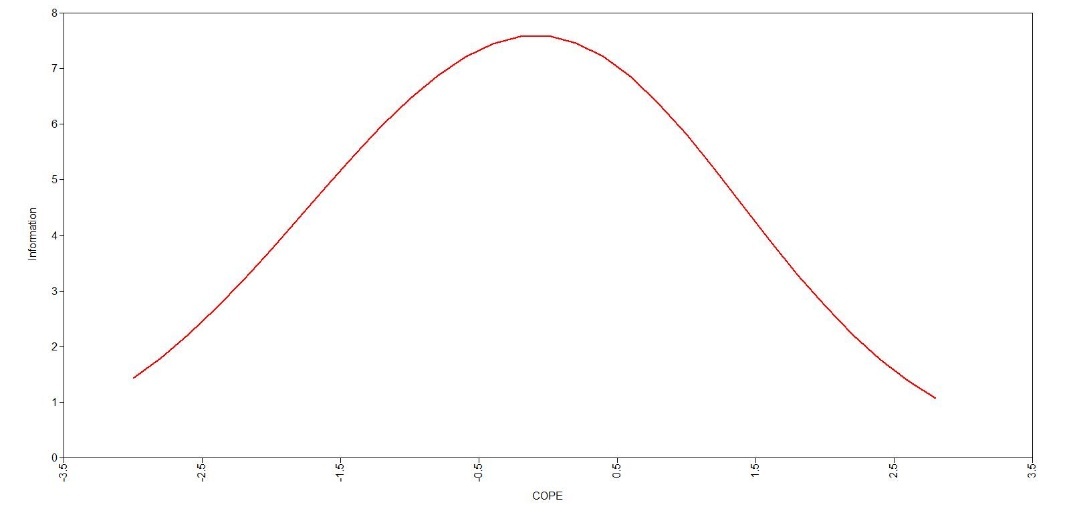**Figure 4a**. Adaptive Coping |  |
| 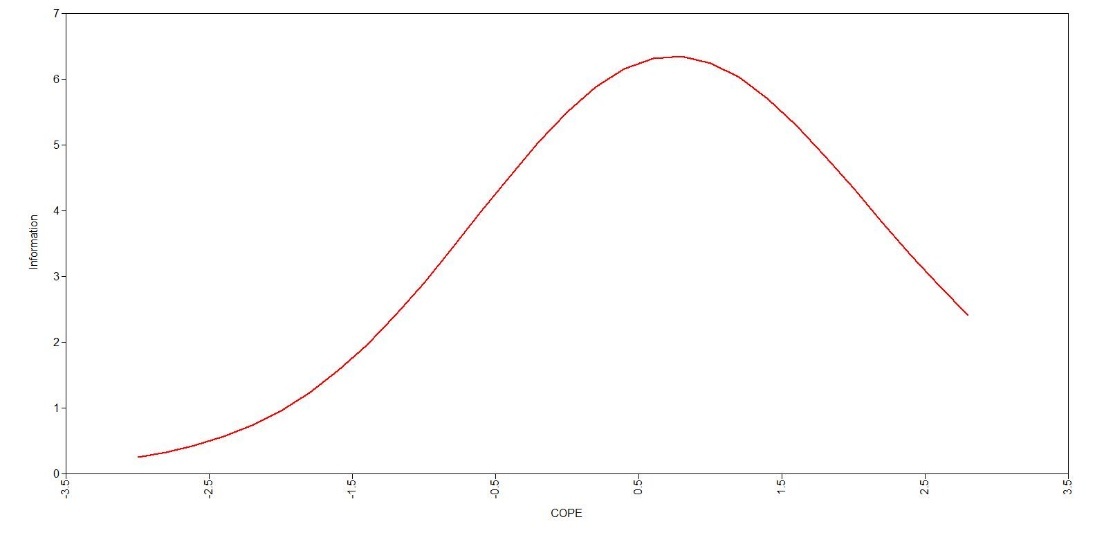  **Figure 4b**. Avoidant Coping  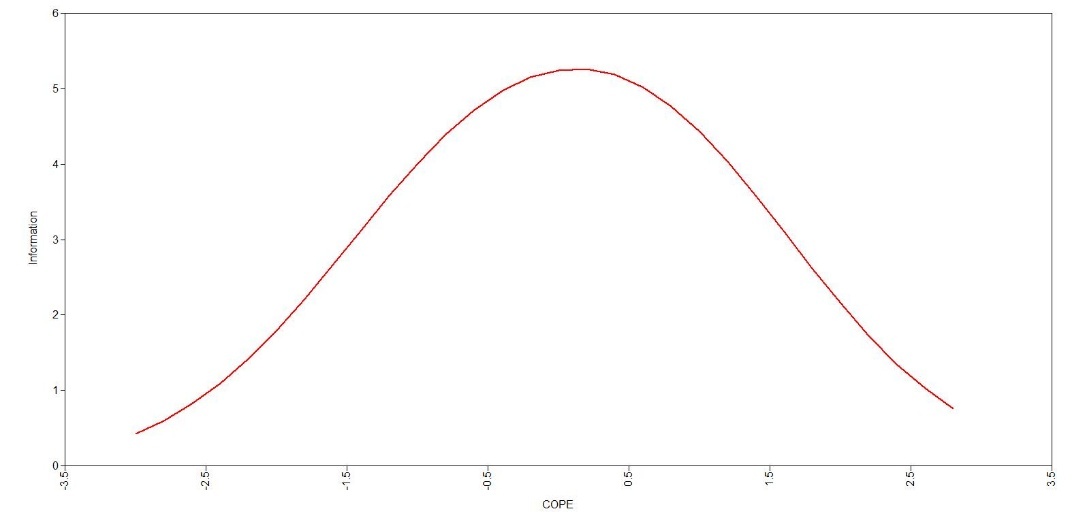  **Figure 4c**. Support Coping |  |
